# Effect of Hyperglycemia on Gene Expression during Early Organogenesis in Mice

**DOI:** 10.1371/journal.pone.0158035

**Published:** 2016-07-19

**Authors:** Jing Zhao, Theodorus B. M. Hakvoort, A. Marcel Willemsen, Aldo Jongejan, Milka Sokolovic, Edward J. Bradley, Vincent C. J. de Boer, Frank Baas, Antoine H. C. van Kampen, Wouter H. Lamers

**Affiliations:** 1 Tytgat Institute for Liver and Intestinal Research, Academic Medical Center, University of Amsterdam, Amsterdam, The Netherlands; 2 Bioinformatics Laboratory, Department of Bioinformatics, Academic Medical Center, University of Amsterdam, Amsterdam, The Netherlands; 3 Department of Biochemistry, Academic Medical Center, University of Amsterdam, Amsterdam, The Netherlands; 4 Department of Genome Analysis, Academic Medical Center, University of Amsterdam, Amsterdam, The Netherlands; 5 Department of Laboratory Genetic Metabolic Diseases, Academic Medical Center, University of Amsterdam, Amsterdam, The Netherlands; 6 Biosystems Data Analysis Group, University of Amsterdam, Amsterdam, The Netherlands; Virgen Macarena University Hospital, School of Medicine, University of Seville, SPAIN

## Abstract

**Background:**

Cardiovascular and neural malformations are common sequels of diabetic pregnancies, but the underlying molecular mechanisms remain unknown. We hypothesized that maternal hyperglycemia would affect the embryos most shortly after the glucose-sensitive time window at embryonic day (ED) 7.5 in mice.

**Methods:**

Mice were made diabetic with streptozotocin, treated with slow-release insulin implants and mated. Pregnancy aggravated hyperglycemia. Gene expression profiles were determined in ED8.5 and ED9.5 embryos from diabetic and control mice using Serial Analysis of Gene Expression and deep sequencing.

**Results:**

Maternal hyperglycemia induced differential regulation of 1,024 and 2,148 unique functional genes on ED8.5 and ED9.5, respectively, mostly in downward direction. Pathway analysis showed that ED8.5 embryos suffered mainly from impaired cell proliferation, and ED9.5 embryos from impaired cytoskeletal remodeling and oxidative phosphorylation (all P ≤ E-5). A query of the Mouse Genome Database showed that 20–25% of the differentially expressed genes were caused by cardiovascular and/or neural malformations, if deficient. Despite high glucose levels in embryos with maternal hyperglycemia and a ~150-fold higher rate of ATP production from glycolysis than from oxidative phosphorylation on ED9.5, ATP production from both glycolysis and oxidative phosphorylation was reduced to ~70% of controls, implying a shortage of energy production in hyperglycemic embryos.

**Conclusion:**

Maternal hyperglycemia suppressed cell proliferation during gastrulation and cytoskeletal remodeling during early organogenesis. 20–25% of the genes that were differentially regulated by hyperglycemia were associated with relevant congenital malformations. Unexpectedly, maternal hyperglycemia also endangered the energy supply of the embryo by suppressing its glycolytic capacity.

## Introduction

Maternal diabetes is a well-established risk factor for congenital malformations in humans [1]. Among these, neural-tube and heart defects, kidney dysgenesis and the caudal regression syndrome are often reported [2–6]. The highest relative risk for major neural tube and cardiovascular defects occurs if the mother develops insulin resistance in the 1^st^ trimester [7,8]. Similar phenomena have been reproduced in rodent models of diabetic pregnancy [9,10]. The animal studies showed that altered expression of genes that regulate the migration of neural crest cells and neural plate closure resulted in patterning defects of the developing head, neural tube and heart [11,12]. *In vitro* studies further showed that a high glucose concentration impaired the proliferation and cell-fate specification of neural stem cells [13].

In mice, ED7.5 appears to be the most sensitive time window for inducing congenital malformations of the neural tube: hyperglycemia at solely this time point suffices to induce these malformations [14,15]. Probably because congenital malformations associated with diabetic embryopathy manifest themselves only on ED10.5, inventories of hyperglycemia-induced changes in gene expression in the embryo were established on ED10.5 [16,17], ED11.5 [18], and between ED13.5 and ED15.5 [19]. These studies showed that maternal hyperglycemia affected the expression of genes involved in apoptosis, proliferation, migration and differentiation during organogenesis in the offspring. It is, however, conceivable that these inventories describe the sequels rather than the targets of the hyperglycemia-induced disturbance in metabolism, because neural-tube formation and neural-crest migration to the heart are initiated during the 8^th^ embryonic day, that is, much earlier [20,21]. We, therefore, analyzed gene expression profiles in embryos of diabetic and nondiabetic pregnancies on ED8.5 and 9.5, that is, shortly after the embryos became sensitive to the hyperglycemia.

Our mouse model of diabetic pregnancy is based on that developed by Loeken [11]. In this elegant model, a moderate dose of streptozotocin (STZ) is used to induce diabetes. After 4–6 weeks of treatment, female mice were exposed to male mice. Because STZ has a very short half-life at neutral pH [22] and because oocytes do not replicate their DNA before fertilization, STZ itself probably has no mutagenic effects on the offspring. Moreover, the effects of STZ can be largely annulled if the STZ-treated mice are also treated with the immunosuppressive drug mycophenolate mofetil [23], suggesting that STZ induces an autoimmune response rather than cytotoxicity. Since the females are not yet severely hyperglycemic at conception, the protocol also avoids the adverse effects of severe maternal diabetes on the growth and maturation of preovulatory oocytes and preimplantation embryos [24]. In fact, severe hyperglycemia develops only after implantation of the embryos at ED4.5, which mimics pregnancy-induced diabetes in humans.

We made an inventory hyperglycemia-induced changes in gene expression in the embryo by creating a Serial Analysis of Gene Expression (SAGE) library and quantifying the mRNA distribution with SOLiD SAGE sequencing [25,26]. Bioinformatic analyses were then used to identify differentially expressed genes to delineate highly regulated pathways and cell-biological processes that were associated with diabetic embryopathy and, finally, to test whether the identified genes and pathways were, if deficient, responsible for neural tube or cardiovascular malformations. We found that that the expression of genes involved in the regulation of cell proliferation, cytoskeletal remodeling, and energy metabolism were most severely affected in ED8.5 and 9.5 embryos. Among the affected genes, many were previously shown to be responsible for neural tube or heart develop, and often for both.

## Materials and Methods

### Animals

FVB mice (9–11 weeks old) were obtained from Harlan Sprague Dawley (Venray, The Netherlands) and fed a diet that was based on Purina 9F (http://www.labdiet.com/cs/groups/lolweb/@labdiet/documents/web_content/mdrf/mdi4/~edisp/ducm04_028438.pdf; production: ABDiets, Woerden, The Netherlands). Mice were kept in groups of 4–5 mice in open cages at the animal facility, on a 12-h light/12-h dark cycle at 22°C with free access to water and food. The study was carried out in accordance with the Dutch Guidelines for the Care and Use of Laboratory Animals and approved by the Ethical Committee for Animal Research of the University of Amsterdam (ALC101225).

### Induction of diabetes

The pregnant diabetic mouse model was adopted from Loeken *c*.*s*. [11]. This model has been extensively evaluated with respect to the malformations it induces in the central-nervous (CNS) and cardiovascular systems of the offspring. Diabetes was induced as recommended by the Animal Models of Diabetic Complications Consortium (AMDCC; [27]). Animals to be treated with streptozotocin were randomly chosen. The diabetic group included 33 and the control group 16 mice. 4–5 diabetic and control mice were housed in one cage. Prior to treatment, female mice were fasted for 6 hours. Sixty mg/kg streptozotocin (STZ; Sigma, Zwijndrecht, The Netherlands), freshly dissolved in 0.1 M sodium citrate (pH 4.5), was injected intraperitoneally for 5 successive days. Mice were screened for glucosuria with KETO-DIABUR-TEST 5000 test strips (Roche, Almere, The Netherlands) from 6 days after the first injection of STZ onwards. Body weight was checked twice weekly. Blood glucose levels were measured before the first injection of STZ and 3 weeks thereafter with a Glucometer Elite (Bayer, Mijdrecht, The Netherlands). Hyperglycemia was treated with subcutaneous sustained-release insulin implants (“linbits”; LinSHIN, Toronto, Canada) that release ~0.1 U/day*implant for at least 30 days. Depending on whether the concentration of blood glucose was 20–25, 25–30, or >30 mmol/L, 1, 1.5, or 2 linbits were implanted. Thereafter, blood glucose was measured weekly. STZ-treated female mice lost ~10% of their body weight in the first 3 weeks after treatment, but regained it upon treatment with the insulin implants (S1 Fig). STZ-treated mice without insulin supplementation were not included in the study. Two-to-three weeks after linbit implantation, mice that maintained glucose levels within the 10–15 mmol/L range were mated with nondiabetic male FVB mice. Noon of the day on which a copulation plug was found was set as embryonic day (ED) 0.5. Only pregnant females with blood glucose levels >17 mmol/L at ED7.5 were included in the experiment (~15 mmol/L is a critical value to induce hyperglycemic damage in embryos [15,28,29]). Pregnant mice were anesthetized with CO_2_ and then sacrificed by cervical dislocation and embryos were recovered between ED7.5 and ED11.5. The developmental stage of the embryos was checked with the criteria of Kaufman [30] (see S1 Table). Only embryos at the proper developmental stage were included in the study.

### Quantification of mRNAs by SOLiD SAGE sequencing

Since we did not have an estimate of the variance in gene expression in our experimental animals, we relied on an approximation [31]. Based on a power of 0.8 and a false discovery rate (FDR) of 0.05, ≥8 arrays, that is, ≥8 embryos per time point and treatment should give informative results [31]. Therefore, 8 male diabetic and 8 male control embryos were randomly chosen from the collection for RNA isolation and SAGE analysis. Total RNA was extracted from the anterior half of embryos (transverse cut made just caudal to the heart) on ED8.5 and from the thoracic segment on ED9.5 (transverse cut made anteriorly between head and thorax, and posteriorly just caudal to the heart), using the TriPure Isolation Reagent (Roche, Almere, The Netherlands). Embryos were frozen and thawed 3 times using liquid nitrogen. After centrifugation, the organic phase of the TriPure extract was re-extracted 3 times. The combined aqueous phases were extracted with choloroform:isoamyl alcohol (24:1) and precipitated with ethanol after addition of 10 μg glycogen. The quantity of total RNA was determined with Qubit 1.0 Fluorometer (Invitrogen, The Netherlands), while the quality was assessed with the Bioanalyser RNA 6000 Nano Chip (Agilent Technologies, Amstelveen, The Netherlands). The RNA integrity number (RIN) of the samples was 9.0–10.0. The total RNA yield per embryo was 0.8±0.1 μg on ED8.5 and 1.8±0.3 μg on ED9.5.

The relative abundance of individual mRNAs was determined by the SOLiD SAGE approach. A double-stranded cDNA library was prepared from at least 0.5 μg RNA, using the SOLiD SAGE Sequencing kit with the Barcoding Adaptor Module (Applied Biosystems, Bleiswijk, The Netherlands; catalog #4452811) and the “working with small amounts of total RNA (1–2 μg)” protocol [26]. Polyadenylated mRNAs were captured with oligo(dT) Dynabeads (Invitrogen; supplied in the kit) and converted to cDNA while attached to the beads. After second-strand synthesis, the cDNA was cleaved with NlaIII. An adapter containing an EcoP15I-recognition site was ligated to the 5’end of the fragment still attached to the bead. EcoP15I cleaves DNA 25–27 bp downstream and releases a tag with a 2-bp overhang. After ligating another adaptor to this overhang, the tag was amplified as described (http://tools.lifetechnologies.com/content/sfs/manuals/cms_084611.pdf). This protocol generates only 1 read per mRNA molecule.

The amplified cDNA libraries, each with a specific barcode introduced during PCR amplification, were pooled in equal ratios. Size and concentration of the libraries were determined using the Bioanalyser DNA-1000 or high-sensitivity chip (Agilent Technologies, Amstelveen, The Netherlands). After affinity selection on beads, the pooled libraries were amplified by emulsion PCR to >30,000 copies, followed by further enrichment on beads and purification as described in SOLiD^™^ 4 System Templated Bead Preparation Guide. High-throughput sequencing was carried out on a SOLiD^™^ 5 system (Applied Biosystems, Bleiswijk, The Netherlands). The sequencing data are available at NCBI (accession number: PRJNA275285; SRA study: SRP056150).

To generate pure reads for analysis, adaptors were removed with Pyrodigm trimming [32]. Alignment was done with the “Burrows-Wheeler aligner” (BWA) against the *Mus musculus* reference genome “mm10”. More specifically, the BWA-backtrack algorithm, which is designed for sequence reads up to 100bp, was used [33]. The DESeq2 package (available through Bioconductor [34]) was used to analyze differential gene expression. Normalization for number of reads per embryo was performed using the “Estimate Size Factors” in the DESeq2 package. P-values were adjusted for multiple testing using the Benjamini-Hochberg procedure. A P_adj_ < 0.05 was considered statistically significant. Clusters were generated with generalized linear models (GLM) based on expression patterns of top 100 genes ranked by P value. Pathway analysis with MetaCore^™^ software (GeneGo, Inc., St. Joseph, MI, USA) was used to assess the significance of changes in gene expression in specified pathways [35,36]. The significance was based on the degree of overlap between the user’s dataset and a set of genes corresponding to a network or pathway queried, assuming the probability that randomly obtained overlaps of a certain size between the user’s set and a network or pathway follow a hypergeometric distribution [37]. MetaCore^™^ determines the degree of association of uploaded datasets with predefined pathways and expresses the similarity in P-values, with lower P-values being more relevant.

### Quantitative PCR analysis of gene expression

Quantitative PCR was performed using a Lightcycler 2.0 with the Fast Start DNA Master^plus^ SYBR Green I kit (Roche, The Netherlands). Primers were designed by the Primer-BLAST tool from NCBI and supplied in S2 Table. cDNA synthesis were performed by gene-specific priming with 40 pmol gene-specific primer and 20 pmol 18S primer. cDNA was diluted 40 times before assaying mRNA expression and 1,000-fold more for 18S expression. Gene expression was normalized relative to the expression of *Tor3a* and *18S*. *Tor3a* was selected as reference genes with the NormFinder software [38] from the SAGE expression data. The average expression of *Tor3a* and *18S* was calculated for each of the genes separately and put at 100% before their relative presence in each of the samples was used for normalization.

### Sex identification

DNA from the visceral yolk sacs of ED8.5 embryos, or the posterior part (cut made just caudal to the heart) of ED9.5 embryos was isolated with TriPure Isolation Reagent (Roche, Almere, The Netherlands). The tissues were taken from the remaining parts of the embryos after harvesting the anterior part (ED8.5) and heart-containing segment (ED9.5) for mRNA quantification (next section). The sex of the embryos was determined by a multiplex PCR amplification using primers to amplify the male-specific *Sry* gene and the autosomal *Il3* gene for reference [39].

### Rate of oxygen consumption and glycolysis

Real-time rates of cellular oxygen consumption (respiration) and proton excretion (glycolysis) in control and hyperglycemic ED9.5 embryo cells were determined using the Seahorse Extracellular Flux (XF-96) analyzer (Seahorse Bioscience, Billerica, MA). 5*10^4^ freshly isolated embryonic cells were seeded in an XF microplate and cultured for 2 hours in the presence of 11.1 mmol/L D-glucose and 10% fetal bovine serum prior to measurement of the oxygen consumption rate (OCR) and extracellular acidification rates (ECAR). Sequential additions of the ATP synthase inhibitor oligomycin, the uncoupling ionophore carbonyl cyanide-p-trifluoromethoxyphenylhydrazone (FCCP), and the cytochrome-c-reductase inhibitor antimycin A were as per the manufacturer’s instructions and allowed determination of the basal rate of oxygen consumption (no additions), the rate of oxygen consumption linked to ATP production (oligomycin addition), the maximal respiration capacity (FCCP addition), and the rate of non-mitochondrial oxygen consumption (antimycin A). The ECAR was determined under basal conditions (no additions) and after inhibition of mitochondrial ATP synthesis with oligomycin. The number of cells was estimated as protein content, which was determined for each well using a standard BCA protein assay. OCR and ECAR values are normalized to mg protein and expressed as the mean ± SEM.

### Data analysis

Gene expression is shown as mean ± SEM. The tests of glucose level and oxygen consumption and extracellular acidification measurements were performed by ANOVA or Student’s t-test. A P < 0.05 was considered statistically significant and < 0.1 as indicating a trend.

## Results

### 1. The mouse model of diabetic pregnancy

**Fig 1:**
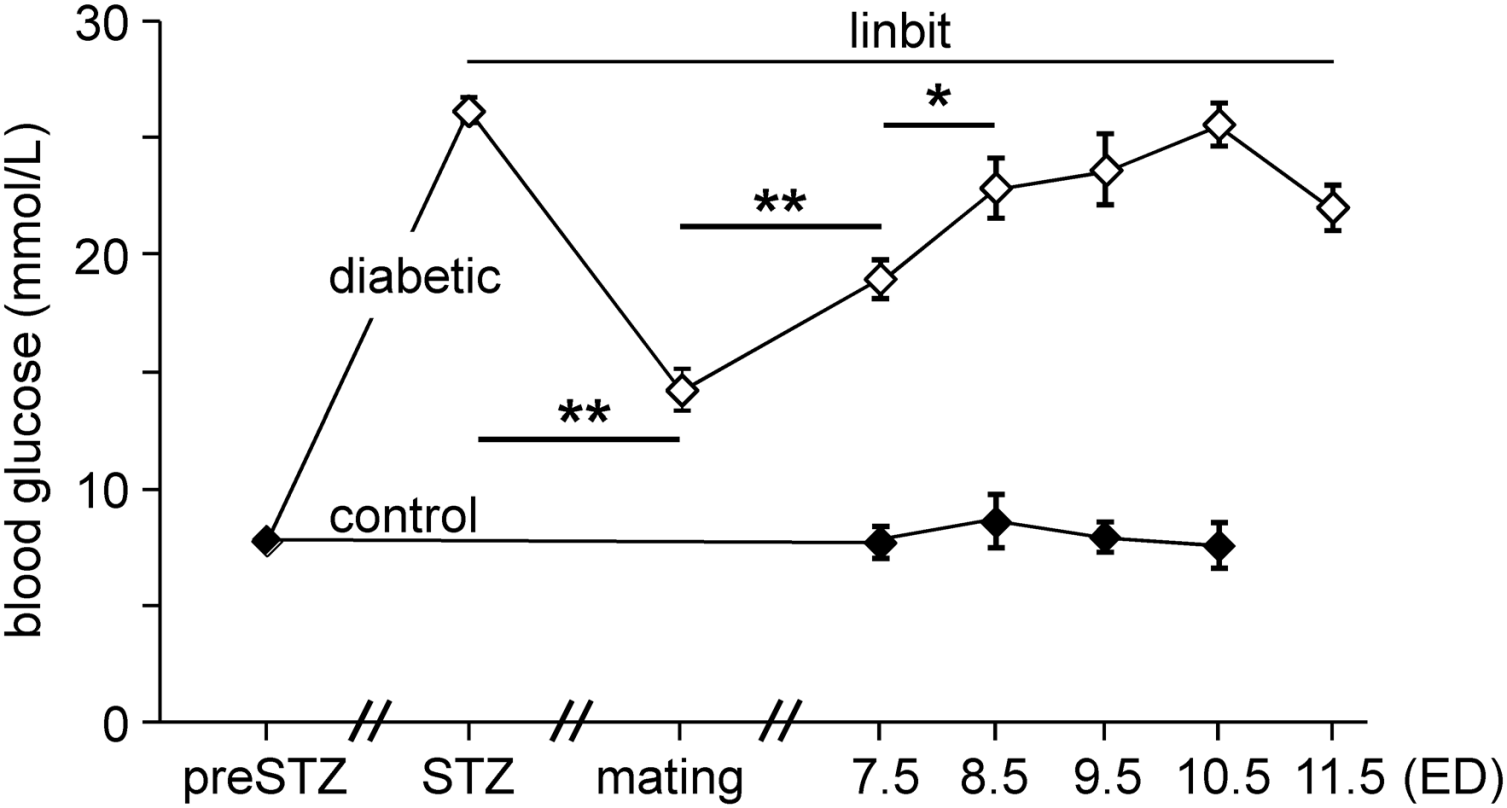
Blood glucose levels in pregnant diabetic mice. Blood glucose levels were measured before streptozotocin (“preSTZ”) treatment, 3 weeks after the first STZ injection (“STZ”), on the day of mating (“mating”), on ED7.5 of pregnancy (“ED7.5”), and at sacrifice (“ED8.5-ED11.5”). After STZ treatment, blood glucose of diabetic mice increased to ~26 mmol/L. Insulin pellets (“linbit”) were implanted after establishing the diabetic state of the mice. Mating was performed when blood glucose was ~14 mmol/L. The control group was not treated with STZ or insulin. Differences between blood glucose levels of diabetic (n = 33) and control mice (n = 16) were analyzed by one-way ANOVA and *t* test. Filled symbols: non-diabetic control mice; open symbols: STZ-treated diabetic mice. **: P < 0.001 (comparison of blood glucose between STZ and mating, mating and ED7.5). *: P < 0.03 (ED7.5 vs ED8.5; ED8.5 did not differ from ED9.5-ED11.5).

In this study we investigated the effects of hyperglycemia on embryos in the early somite period, that is, on ED8.5 and ED9.5 (7–20 somites). The blood glucose levels in the dams from the induction of diabetes to sacrifice are shown in Fig 1. Blood glucose concentrations of female mice after induction of diabetes (P < 0.001), during the mating period (P < 0.01), and during pregnancy (P < 0.001) were significantly higher than those in controls. In diabetic mice, maternal blood glucose concentrations decreased to ~50% after the implantation of sustained-release insulin implants (“linbits”; P < 0.001), while maternal blood glucose concentrations rose markedly between mating and ED7.5 (P < 0.001) and between ED7.5 and 8.5 (P < 0.03), after which it plateaued (P_8.5*vs*9.5_ = 0.72, P_9.5*vs*10.5_ = 0.38 and P_10.5*vs*11.5_ = 0.28). Because human clinical studies showed that male offspring was more sensitive to the effect of maternal diabetes than female [40], male mouse embryos were investigated. All embryos in the study were alive and none was externally abnormal or delayed in development (S2 Fig).

### 2. Global profile of gene expression in ED8.5 and 9.5 embryos

To obtain a comprehensive overview of the response of embryos to maternal hyperglycemia at an early developmental stage, whole transcriptome profiles of ED8.5 and ED9.5 embryos were investigated with SOLiD SAGE mRNA deep sequencing. To obtain a representative expression-profile library, construction had to be started with at least 0.5 μg mRNA to assure that the number of detected genes for a library was not dependent on the input of total RNA (S3 Fig). We obtained ~8*10^6^ pure reads and the expression profile of ~2*10^4^ genes per embryo.

**Fig 2:**
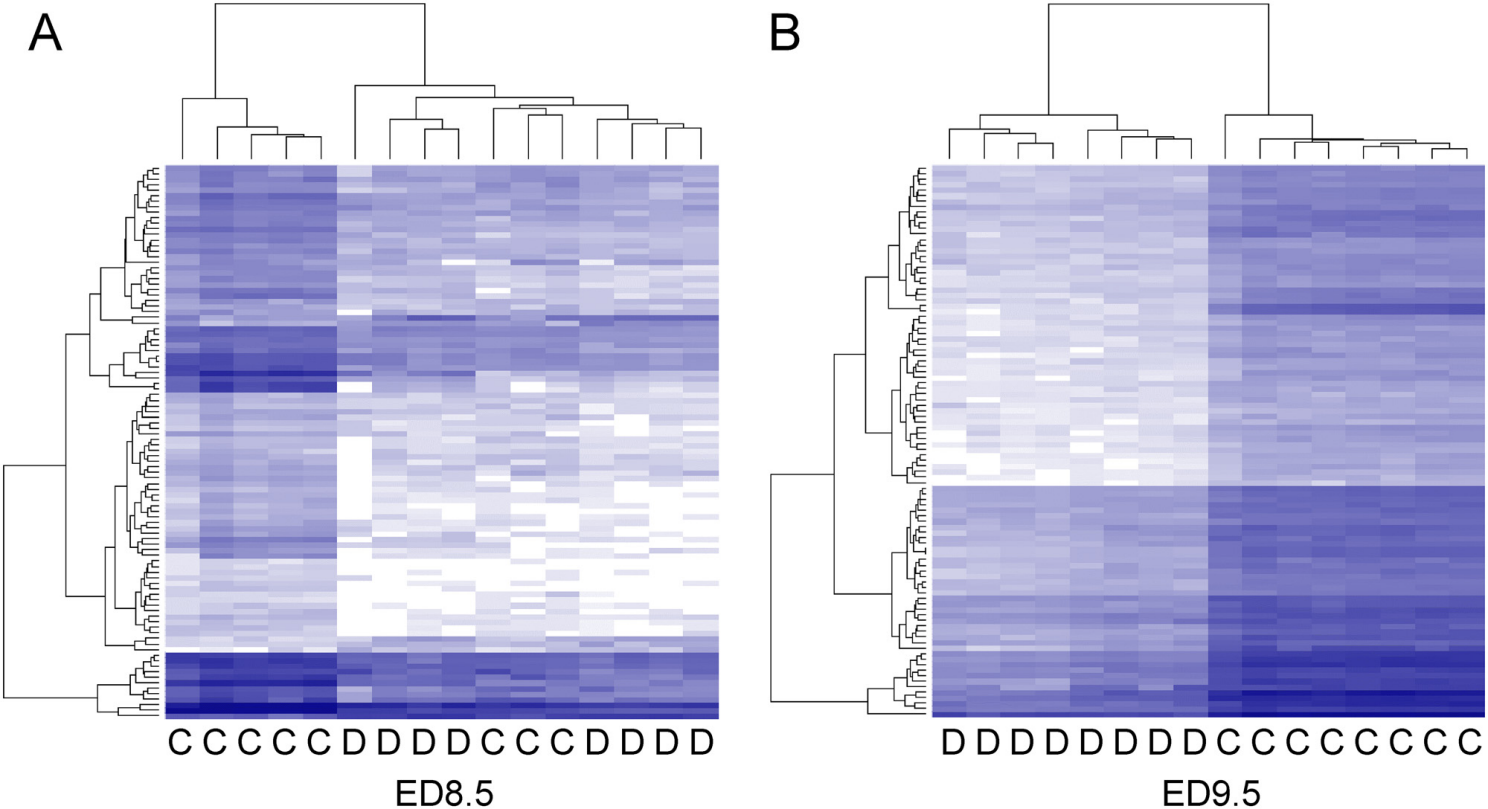
Hierarchical clustering of the changes in gene expression in control and hyperglycemic ED8.5 and ED9.5 embryos. Messenger RNA from control and hyperglycemic ED8.5 (left panel) and ED9.5 embryos (right panel) was extracted (8 embryos per group) and each used for the construction of a separate SOLiD SAGE library. “C” and “D” at the bottom margin of the panels indicate “control” and “diabetic” (hyperglycemic) embryos, respectively. Hierarchical clustering was performed by DESeq analysis. The dendrogram on the top of the panels reflects the correlation in differential gene expression between embryos, with less height indicating a higher similarity. The dendrograms at the left margin indicate the correlation in the response of the respective genes. The intensity of the blue color represents the relative level of expression of a gene (after transforming the data for variance stabilization). One ED8.5 hyperglycemic embryo was removed because the input of mRNA was low (< 0.5 μg) and subsequent output of mRNA too small (see S1 Fig). Three ED8.5 control embryos clustered with the hyperglycemic embryos and were not included in the analyses (see main text). At ED8.5, 2305 genes were differentially expressed (panel A; 7 hyperglycemic and 5 control embryos were compared). On ED9.5, 4640 were differentially expressed (panel B).

**Fig 3:**
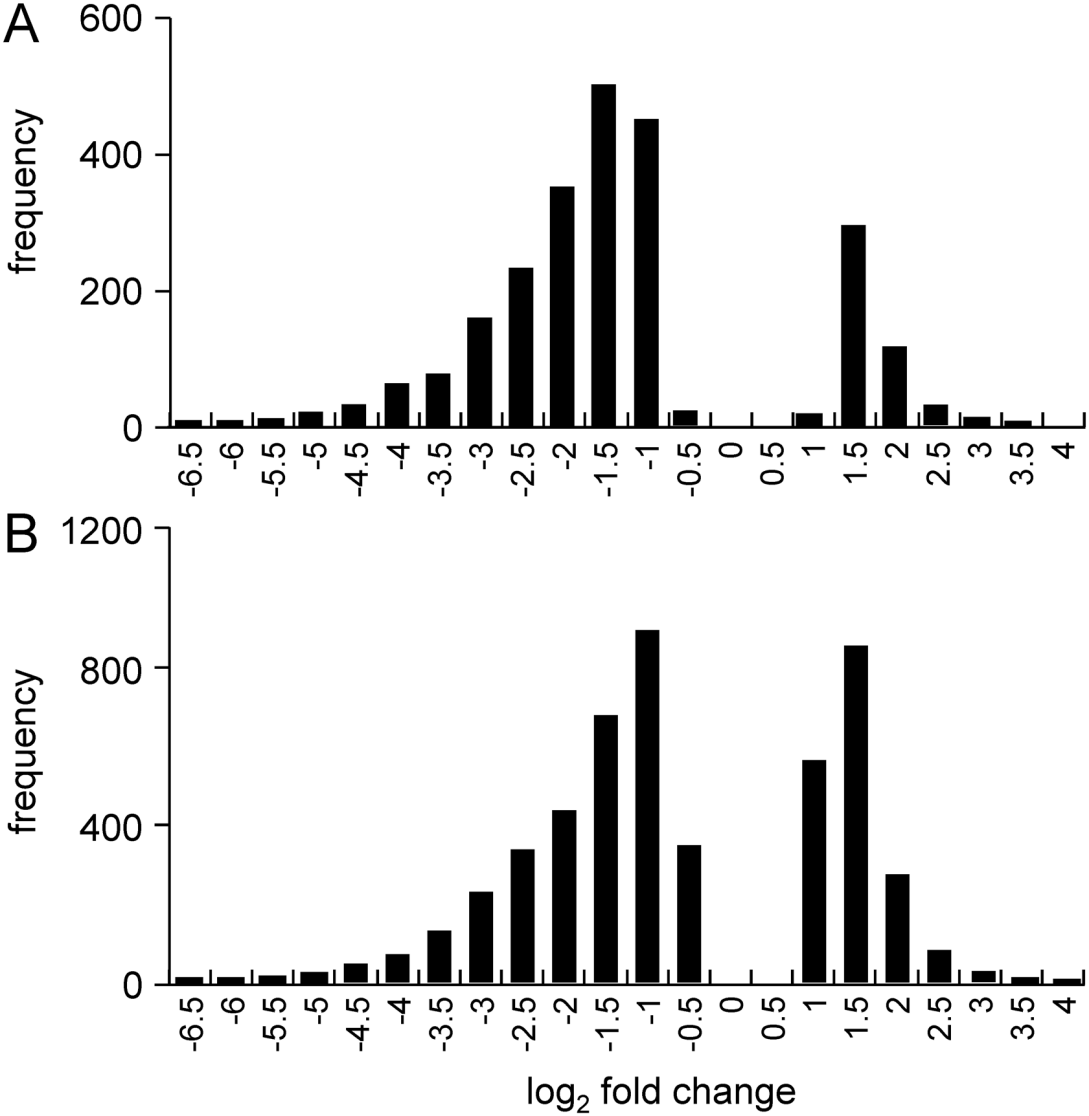
The distribution of the fold-change of differentially expressed genes in ED8.5 and 9.5 embryos. Panels A and B show the fold-change of the 2305 and 4640 differentially expressed genes on ED8.5 and 9.5, respectively, with fold change ≥1.5, as detected after DESeq analysis. The X-axis shows bins with log_2_-fold change, while the Y-axis indicates the frequency of each event. Note that the majority of genes were downregulated in hyperglycemic embryos (~80% on ED8.5 and ~65% on ED9.5).

**Fig 4:**
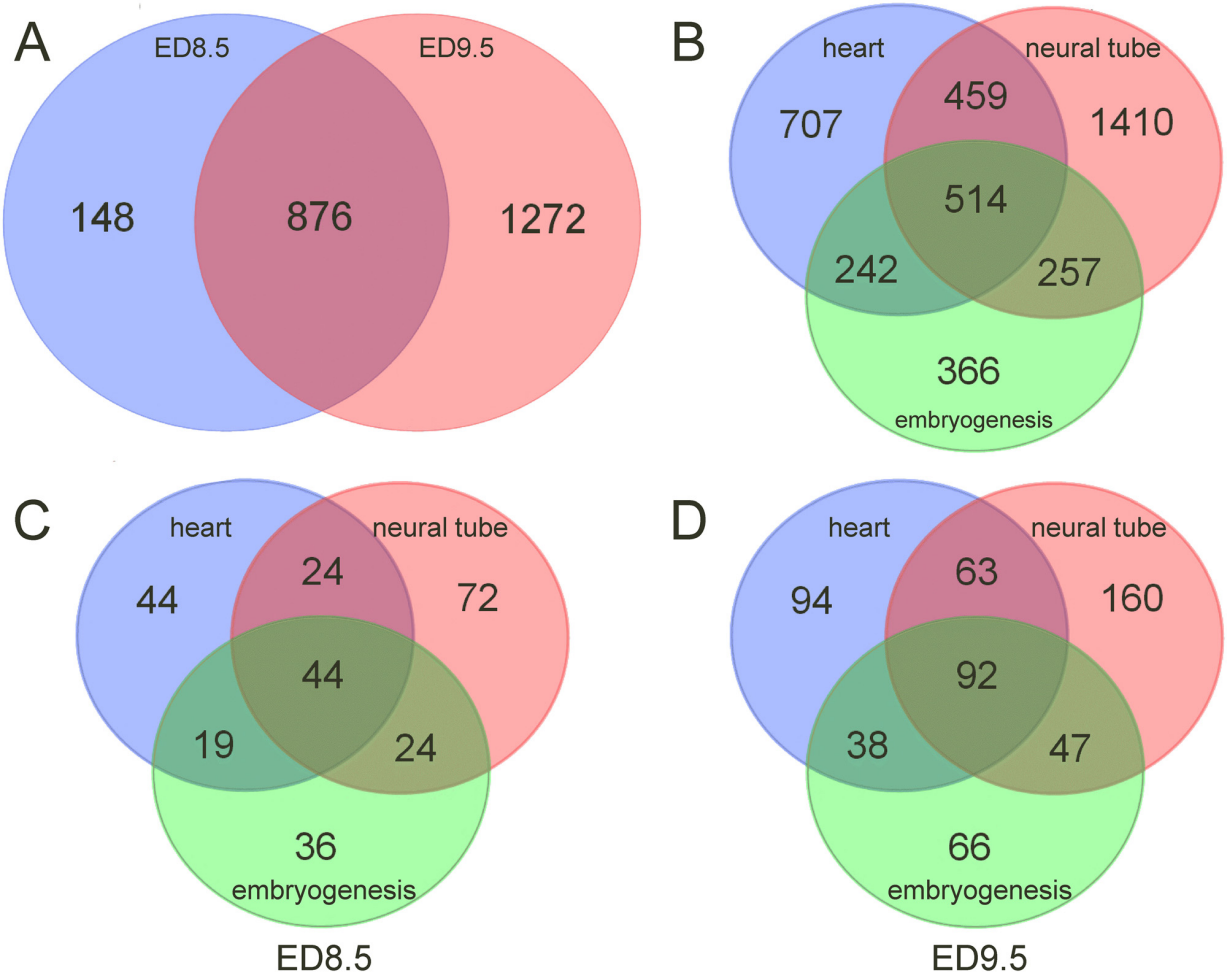
Genes differentially expressed in hyperglycemic ED8.5 and ED9.5 embryos associated with cardiovascular and/or neural defects. Panel A: 1024 and 2148 were differentially expressed, unique functional genes in embryos from hyperglycemic and control dams on ED8.5 and 9.5, respectively, 876 of which were common to ED8.5 and ED9.5. The Jackson Laboratory Mouse Genome Database (MGD) at the Mouse Genome Informatics website (URL:http://www.informatics.jax.org; October 2014) was queried by selecting “Phenotypes & Mutant Alleles—Phenotypes, Alleles & Disease Models Query—Anatomical Systems Affected by Phenotypes” and searching with “Cardiovascular system”, “Nervous system” and “Embryogenesis” as key words (total number of genes retrieved: 3955). Panel B shows a Venn diagram of the number of genes associated with the respective categories in the MGD database. Of these genes, 131, 164 and 123 among the 1024 differentially expressed genes on ED8.5 (panel C), and 287, 362 and 243 out of 2148 genes on ED9.5 (panel D) were associated with cardiovascular, neural or embryogenesis defects, respectively.

The dendrogram generated by non-supervised hierarchical clustering with correlation as similarity measure and average linkage as clustering parameter revealed that maternal diabetes separated control and hyperglycemic groups into distinct branches on ED9.5 (Fig 2B), but not yet on ED8.5 (Fig 2A). The length of a branch reflects the degree of difference between samples. Of the 8 control and 8 hyperglycemic ED8.5 embryos that were analyzed, a subgroup of 3 control embryos clustered with the hyperglycemic embryos (Fig 2A). These differently behaving control embryos were from 2 litters that also had a member in the main control group. Furthermore, one hyperglycemic embryo behaved quite differently from the remaining embryos, because the input of mRNA for cDNA synthesis was too small (0.3 μg; no saturation in genes detected, see S3 Fig). To identify the regulated pathways on ED8.5, we performed pathway analysis on the 7 hyperglycemic and 5 control embryos that clustered together. Compared to the control group, 2305 (ED8.5) and 4640 (ED9.5) unique transcripts met our boundary condition for significance (P_adj_ < 0.05) and were ≥ 1.5-fold up- or downregulated as a result of maternal diabetes. The log_2_ fold change distribution of differentially expressed genes (P_adj_ < 0.05) in the entire population on ED8.5 and 9.5 is shown in Fig 3. The majority of these genes were down-regulated (~80% on ED8.5 and ~65% on ED9.5). After removal of RIKEN, EST, unassigned and hypothetical gene sequences, 1024 functional unique genes were changed in expression in ED8.5 and 2148 genes in ED9.5 hyperglycemic embryos (~45% of the original number on both days). Of the genes that were differentially expressed on ED8.5, ~85% was also differentially expressed on ED9.5 (Fig 4A), but represented only ~40% of all differentially expressed genes on that day, implying that the main difference between ED8.5 and ED9.5 was that the number rather than the identity of affected genes had expanded. A list of all differentially expressed genes on ED8.5 and ED9.5, and their fold change, can be found in S3 Table.

**Fig 5:**
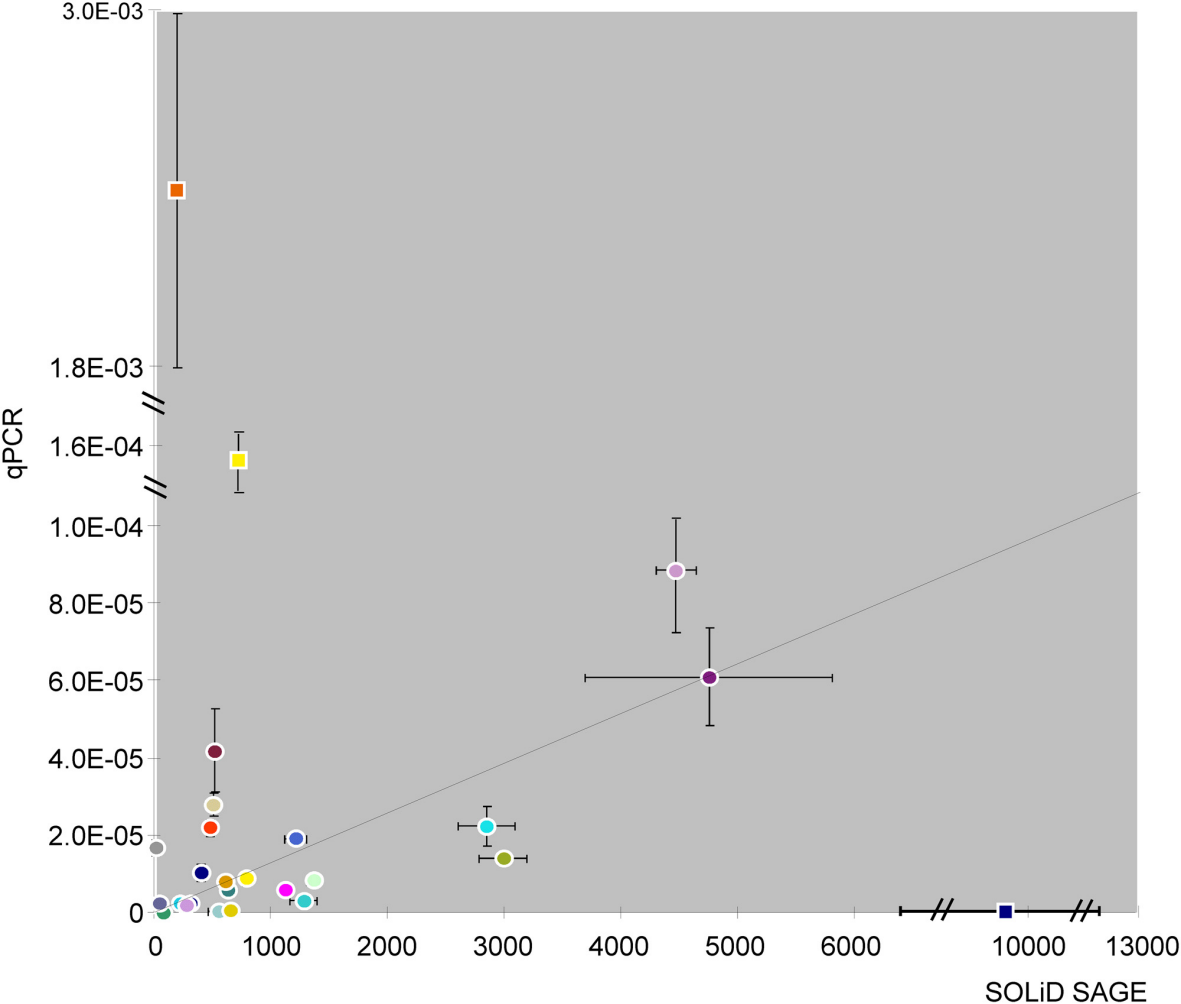
Correlation of mRNA expression levels analyzed by SOLiD SAGE sequencing and quantitative PCR analysis. The mRNA of 8 euglycemic embryos on ED9.5 was quantified by SOLiD SAGE sequencing. Based on P_adj_ (<0.05) and fold-change (>1.5) of differentially expressed genes in SOLiD SAGE sequencing, 11 genes without change, 4 up-regulated genes and 11 down-regulated genes were selected for validation by qPCR. The mRNA of 8 other euglycemic embryos on ED9.5 was quantified by qPCR after cDNA synthesis by gene-specific priming. The X-axis shows the number of reads after SOLiD SAGE sequencing and normalization for total reads in each embryo. The Y-axis indicates gene expression in qPCR normalized by the expression of reference genes *Tor3a* and 18S. The error bar indicates ± SEM.

To validate the SAGE analysis, among 2148 unique differentially expressed genes on ED9.5, 11 down-regulated (*Ndufa6*, *Actg1*, *Glut4*, *Fgf2*, *Tpm4*, *Marcksl1*, *Myosin1H*, *Axin1*, *Mrto4*, *Psmc4* and *Ptpn11*) and 4 up-regulated (*Mrps23*, *Rnaseh2c*, *Cdk1* and *Ubxn8*) genes were selected for validation by quantitative PCR (qPCR). The prevalence ranged from rare to abundant. In addition, 11 genes without change in expression (P_adj_ > 0.05 and fold-change ~1; *Gtf3c2*, *Raf1*, *Tab2*, *Trip13*, *Atp5c1*, *Cdc20*, *Tor3a*, *Mtch1*, *Pax3*, *Bax* and *Arg1*) were selected. Fig 5 shows the correlation of expression levels of these genes between SOLiD SAGE sequencing and qPCR in control embryos (the values and the color code of the symbols are shown in S4 Table). The range of expression across which this correlation was tested was ~10^3^. Apart from 3 obvious outliers (*Cdc20*, *Actg1*, and *Atp5c1*), the expression as assayed by SAGE sequencing and qPCR correlated (R^2^ = 0.57). We have not been able to identify technical errors that could have accounted for the outliers.

### 3. Pathways regulated by maternal hyperglycemia

Since a global analysis does not reveal a direction in the changes and lacks functional detail, we scrutinized the data for biological pathways with functional implications with MetaCore^™^. The 2305 transcripts on ED8.5 and the 4640 transcripts on ED9.5 that were differentially expressed between embryos of hyperglycemic and control mice were analyze for linkage to pathways in the MetaCore^™^ suite. As shown in Table 1, the cell cycle and cell proliferation on ED8.5 (positions 1–3 and 8 on ED8.5; position 4 on ED9.5), cytoskeletal remodeling and cell adhesion on ED9.5 (positions 1, 2, 5, 7, and 9 on ED9.5; positions 4, 5, 7 and 9 on ED8.5), and oxidative phosphorylation (position 3 on ED9.5 and 6 on ED8.5) were very prominent among the top 10 highly regulated pathways that differ between hyperglycemic and control embryos on ED8.5 and 9.5 (all P ≤ E-5). The level of significance was higher on ED9.5, largely because more genes were affected. Taking into account that the cited regulated pathways often shared genes, the cell cycle was more prominently affected in hyperglycemic embryos on ED8.5 than ED9.5 (P value smaller on ED8.5 than ED9.5), whereas cytoskeleton remodeling, together with cell adhesion and migration, and oxidative phosphorylation became more vulnerable on ED9.5 (P value larger on ED8.5 than ED9.5). The suppression of oxidative phosphorylation by maternal hyperglycemia became more important on ED9.5 as deduced from the ~100-fold smaller P value on ED9.5 compared to ED8.5. Four pathways (oxidative phosphorylation, Wnt/TGFβ signaling, cytoskeleton remodeling (ephrin B), and cytoskeleton remodeling (remodeling), were highly regulated in both ED8.5 and ED9.5 embryos. On closer inspection, only 0%, 4%, 12%, and 19% of the 21, 16, 31 and 27 genes, respectively, that were differentially regulated in these pathways at ED8.5, were not present in the same pathways on ED9.5. Because the number of genes affected in these pathways increased 1.5–2.5-fold, this finding indicates that the number of affected genes in these pathways increased between ED8.5 and ED9.5 rather than the identity of the pathways involved.

**Table 1:** Highly regulated pathways in ED8.5 and ED9.5 hyperglycemic embryos.

	Rank	Pathways	P value
ED 8.5	1	Cell cycle _Spindle assembly and chromosome separation	E-8
	2	Development_Flt3 signaling	E-6
	3	Cell cycle_Role of Nek in cell cycle regulation	E-6
	4	Cytoskeleton remodeling_Cytoskeleton remodeling	E-6
	5	Cytoskeleton remodeling_Regulation of actin cytoskeleton by Rho GTPases	E-6
	6	Oxidative phosphorylation	E-6
	7	Cytoskeleton remodeling_Reverse signaling by ephrin B	E-5
	8	Development_IGF-1 receptor signaling	E-5
	9	Cytoskeleton remodeling_TGF, WNT and cytoskeletal remodeling	E-5
ED 9.5	1	Cytoskeleton remodeling_Cytoskeleton remodeling	E-10
	2	Cytoskeleton remodeling_TGF, WNT and cytoskeletal remodeling	E-10
	3	Oxidative phosphorylation	E-8
	4	Cell cycle_Chromosome condensation in prometaphase	E-7
	5	Cell adhesion_Integrin-mediated cell adhesion and migration	E-7
	6	Chemotaxis_CXCR4 signaling pathway	E-6
	7	Cytoskeleton remodeling_Reverse signaling by ephrin B	E-6
	8	Development_EPO-induced Jak-STAT pathway	E-6
	9	Cell adhesion_Chemokines and adhesion	E-6

Pathways that were identified by MetaCore^™^ as highly regulated by maternal hyperglycemia in ED8.5 and ED9.5 embryos. The selection of pathways was limited to those with a P value ≤ E-5.

### 4. Differentially expressed genes are involved in heart and/or neural-tube defects

To establish if the differentially expressed genes on ED8.5 and ED9.5 could account for heart or neural-tube defects if downregulated, we queried the Jackson Laboratory Mouse Genome Database (MGD; for protocol, see the legend of Fig 4). This database contains 3781 genes that, if deficient, cause cardiovascular defects, 5195 genes that cause neural (“cerebrospinal”) defects, and 2746 genes that have general effects on embryonic development (database queried in October 2014; Fig 4B). Among the 1024 genes that were present in the MGD and differentially expressed in hyperglycemic and control embryos on ED8.5, 131, 164 and 123 (6.8, 6.2, and 8.9% in each group) were related to defects in cardiovascular, neural, and general embryonic development (Fig 4C), respectively, while among the 2148 genes that were in the database and differentially expressed on ED9.5, 287, 362, and 243 (14.9, 13.7 and 17.6% in each group) were related to the respective defects (Fig 4D). Of the differentially expressed genes that were linked with cardiovascular or neural-tube defects, 23.3% was associated with both conditions on both ED8.5 and ED9.5 (~13.6% and ~16.9% were exclusively associated with cardiovascular and neural-tube defects, respectively, on both days (S4 Fig)). In total, 26% of the differentially expressed genes in either ED8.5 or ED9.5 hyperglycemic embryos were causally associated, if deficient, with cardiovascular or neural defects according to the MGD database (S5 Fig). Of the genes relevant to each defect that were identified on ED8.5, 85.5% was still present in the group of affected genes on ED9.5 (Fig 4A), again implying that the hyperglycemic condition of the embryos had aggravated their condition between ED8.5 and ED9.5.

It should be kept in mind that the MGD database describes the phenotype after complete inactivation of single genes, whereas a partial polygenic etiology is considered more likely. We, therefore, checked the number of genes that were >5-fold and >10-fold up- or downregulated on ED8.5 and ED9.5, and were associated with cardiovascular or neural malformations if absent (S5 Fig). On ED8.5, 217 and 55 genes were changed >5- and >10-fold, respectively, while on ED9.5, these numbers were 299 and 90, respectively. Of these, 23–24% and 4–5% were, if deficient, associated with congenital malformations on ED8.5 and ED9.5, respectively. A 10-fold reduction is what one reads in published knockdown experiments. Furthermore, the expression of almost all of them (~98%) was suppressed and at least some of the affected genes may well have acted synergistically.

The 106, 138 and 99 genes that were differentially expressed in hyperglycemic embryos on both ED8.5 and 9.5 in cardiovascular, neural and whole-embryonic developmental defects, respectively, were used for pathway analysis in MetaCore^™^ and found to be also mainly involved in cytoskeletal organization, including cell adhesion (P < 5E-5; not shown). This finding underscores that the pathways that were identified as being highly regulated by hyperglycemia in ED8.5 and ED9.5 embryos (Table 1) are very relevant for the malformations in the cardiovascular and neural systems that are observed in diabetic pregnancies. As already stated, a high percentage of these genes affected both cardiovascular and neural development (Fig 4).

### 5. Energy metabolism

**Fig 6:**
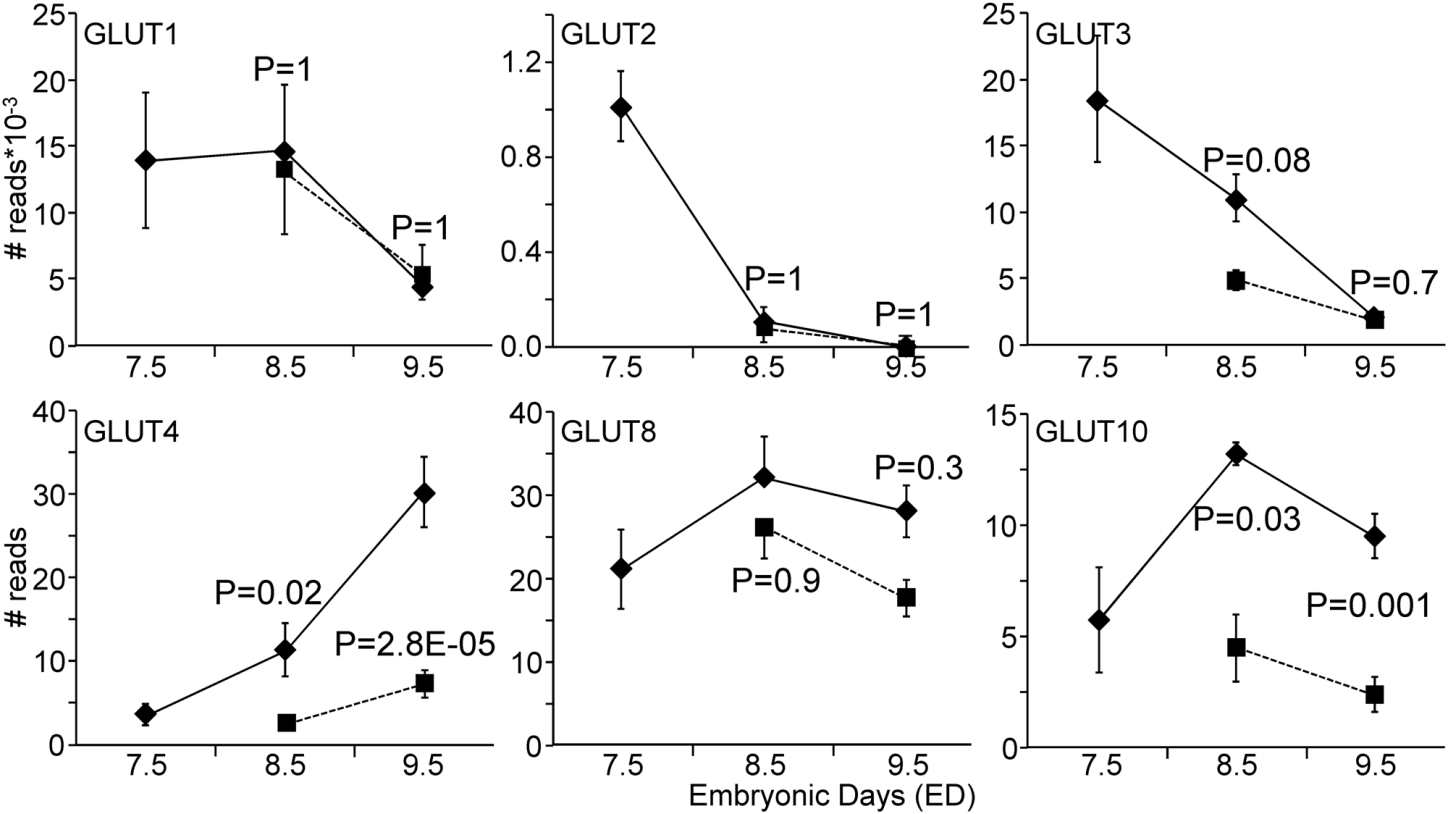
Expression of glucose transporters in control and hyperglycemic embryos. The most highly expressed glucose transporters (*Glut1-3*) all declined during normal development (ED7.5–9.5), whereas the insulin-regulated glucose transporters (Glut4, -8, and *-10*) increased in concentration (note that the *Glut1-3* values are expressed after division by 1,000). Hyperglycemia had no (*Glut1* and *-2*) or a small negative effect (*Glut3* on ED8.5 only) on the highly expressed glucose transporters, but clearly suppressed two of the insulin-regulated transporters. Diamond symbols indicate the control group and square symbols the hyperglycemic group. P values compare expression between hyperglycemic and control embryos on the indicated day.

The expression of genes involved in oxidative phosphorylation was strongly regulated in both ED8.5 and ED9.5 hyperglycemic embryos (Table 1). The expression of subunits of mitochondrial enzymes *Cox8a* (cytochrome c oxidase subunit 8A) and *Atp5e* (ATP synthase subunit) were, for example, >10-fold down-regulated, whereas *Ndufa1* (NADH dehydrogenase subcomplex) was 5-fold up-regulated on ED9.5, all suggesting inhibition of mitochondrial respiration and adaptation to glycolytic flux. We anticipated that hyperglycemia would also affect the expression of glucose transporters. Fig 6 shows that *Glut1*, *-2*, and *-3* (*Slc2a1-3*) were most highly expressed, but all 3 transporters showed a pronounced decrease in expression between ED7.5 and ED9.5, and were not affected by hyperglycemia, except for a trend for *Glut3* (P = 0.08). In contrast, the insulin-responsive glucose transporters *Glut4*, *-8*, and *-10* (*Slc2a4*, *-8*, and *-10*) were expressed to a much lower extent, but increased with development and, with the exception of *Glut8*, were negatively affected by hyperglycemia. *Glut4* was most sensitive in this respect, with a ~5-fold decreased expression in hyperglycemic embryos. The expression of sodium-coupled glucose transporters was extremely low (not shown).

**Fig 7:**
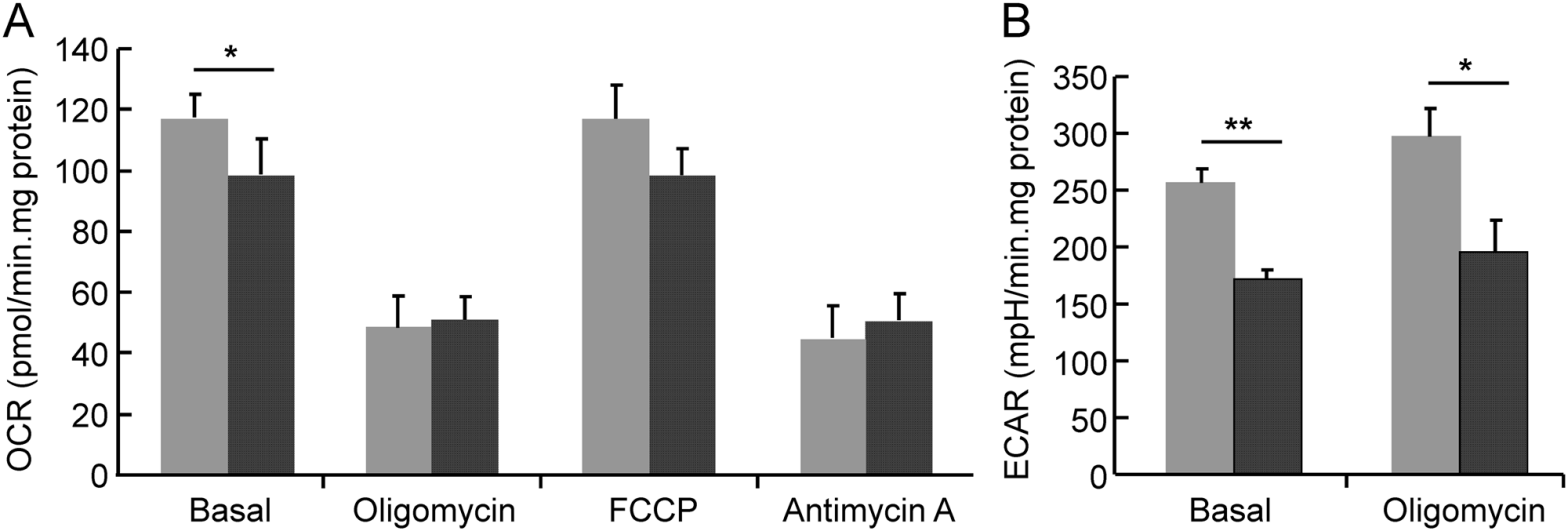
Oxygen consumption and extracellular acidification in cells of control and hyperglycemic embryos on ED9.5. Cells obtained from 12 hyperglycemic and 17 control ED9.5 embryos were used to measure the rate of oxygen consumption (OCR; panel A) and extracellular acidification (ECAR; panel B). Gray bars represent cells from control and black bars from hyperglycemic embryos. The bars indicate the OCR and ECAR under basal conditions and after sequential addition of oligomycin, FCCP and antimycin A. Data represent 3 consecutive measurements of separate dishes with cells from 4 hyperglycemic embryos and 6 control embryos on average. The Y-axis indicates oxygen consumption (pmoles/min.mg protein) or extracellular acidification (mpH/min.mg protein). The asterisk indicates a significant difference between control and hyperglycemic embryos. **: P < 0.01. *: P = 0.03 (basal OCR control vs diabetic) and P = 0.01 (ECAR after treatment of oligomycin control vs diabetic).

To investigate whether these changes in gene expression reflected differences in mitochondrial function and/or glycolysis, we measured the rates of oxygen consumption and extracellular acidification in cells of control and hyperglycemic ED9.5 embryos. As shown in Fig 7, the rate of basal oxygen consumption in diabetic cells was ~85% of that in control cells (P = 0.03). No differences were observed after oligomycin, FCCP, and antimycin A addition, revealing that the maximal respiration capacity (FCCP addition) and the rate of non-mitochondrial oxygen consumption (antimycin A) were not different. Oxidative phosphorylation (basal OCR—oligomycin-suppressed OCR) in cells from control and hyperglycemic embryos were not different (68 ± 11 and 47 ± 10 pmol/min.mg protein, respectively (P = 0.19)). The extracellular acidification rate was significantly lower in cells from hyperglycemic than from control embryos (~70%; P = 0.01). Furthermore, glycolysis already functioned at the maximal rate under basal conditions, as the addition of oligomycin did not increase ECAR. In addition, these data show that ATP synthesis from glycolysis (~25 nmol/min.mg protein) in cells from control embryos is ~150-fold higher than that from oxidative phosphorylation (~0.17 nmol/min.mg protein; phosphate/oxygen ratio set at 2.5). Because both oxidative phosphorylation and glycolysis are ~30% lower in cells from hyperglycemic embryos, the ratio remains similar. These data show that glycolytic activity was the main source of energy production in ED9.5 embryos and that its activity and capacity was substantially lower in hyperglycemic embryos, indicating they had an energy problem.

## Discussion

Earlier studies on ED10.5–15.5 embryos showed that maternal hyperglycemia affects the expression of genes involved in apoptosis, proliferation, migration and differentiation [16–19]. In the present study, we examined the effect of maternal hyperglycemia in mouse embryos during gastrulation and early organogenesis (ED8.5 and ED9.5). The main findings were that pregnancy aggravated hyperglycemia in mildly diabetic dams, as it does in humans [41], and that hyperglycemia in the embryo developed before the onset of insulin production and affected the expression of genes involved in the regulation of cell proliferation, cytoskeleton, and energy metabolism. Approximately 25% of the differentially regulated genes were associated with cardiovascular and neural malformations if deficient.

### Hyperglycemia and glucose transport

Insulin (and glucagon) can be detected only in embryos with >20 somites (ED9.25) [42], and maternal insulin does not reach the embryo proper [43]. In agreement, our gene expression data do not show expression of the insulin-signaling factors *Insr*, *Irs1/2*, or *Akt1* in ED8.5 or ED9.5 embryos (*Pik3* is expressed). Embryonic hyperglycemia, therefore, most likely exerts its teratogenic effects in the absence of insulin, that is, directly on sensitive cells via glucose transporters or as osmolyte.

Although *Glut1*, *-2*, and *-3* are mostly expressed in the extraembryonic tissues (yolk sac, amnion and/or ectoplacental cone) [44,45], expression of *Glut1* and *Glut3* in the embryo was >100-fold and that of *Glut2* >10-fold higher than the other glucose transporters. Expression of these *Gluts* peaked, in agreement with literature [44,46–49], during implantation and gastrulation, and declined in a pronounced manner after ED7.5 (*Glut2*, *Glut3*) or ED8.5 (*Glut1*). Also in agreement with literature [45,50,51], the expression of *Glut1*, *-2*, and *-3* was not or only mildly affected by hyperglycemia. Since *Glut2* deficiency appears to protect the embryo from the teratogenic effects of hyperglycemia [51], the pronounced decline of its expression after ED7.5 may explain the window of sensitivity to hyperglycemia around ED7.5 [15].

In contrast to *Glut1*, *-2*, and *-3*, the expression of *Glut4* and *Glut10* increased with development and was strongly suppressed by hyperglycemia. Expression of *Glut4* in postimplantation embryos first appears in the yolk sac and ectoplacental cone, and then increasingly also in the embryonic tissues [48,52]. GLUT4, -8 and -10 are all recruited to the plasma membrane via an insulin-dependent increase in cytoskeletal actin polymerization [53–55] and become expressed only when endogenous insulin production starts at ED9.5 [42]. The phenotype of *Glut4*- [56], *Glut8*-[57], or *Glut10*-deficient [58] embryos does not suggest a role for these transporters in hyperglycemic embryopathy, but it remains to be established whether the deficiency protects against the adverse effects of hyperglycemia in early development [59].

### Hyperglycemia and the cytoskeleton

The cytoskeleton plays a prominent role in the changes in cell shape and the morphogenetic movements that characterize cell behavior during gastrulation and organogenesis [60,61]. Hyperglycemia suppresses the cell cycle [62] and causes a pronounced downregulation of the expression of many genes of key components of the cytoskeleton in embryos. Embryonic hyperglycemia suppressed the expression β- and especially γ-cytoskeletal actin, the cobblestones of the dynamic submembranous cytoskeletal network, ~3- and ~100-fold, respectively. The expression of associated small GTPases, including *Rho*, *Cdc42*, and *Rac*, and the *ezrin*, *radixin*, and *moesin* (ERM) proteins [63,64] was also affected. Exposure to glucose phosphorylates and activates Cdc42. Interaction of active Cdc42 with WASP receptors activates ARP2/3, which enhances actin nucleation and branching. Activated ARP 2/3, in turn, joins onto pre-existing microfilaments and creates a site for the extension of new microfilaments (branching). Cofilin, another downstream signal of Cdc42 and other members of the RHO family that is active in its dephosphorylated form, promotes actin depolymerization. Integrins or PKC are major activators of cofilin-1. The balance of N-WASP and cofilin activities determines the direction of cytoskeleton dynamics. Since the expression of virtually all these genes was affected, cytoskeletal dysfunction must be a prominent sequel of embryonic hyperglycemia. Hyperglycemia also enhances the depolymerization and polymerization cycle rate (“dynamic instability”) of cytoskeletal actin [63,65,66]. This post-translational effect of hyperglycemia appears to be mediated by the hyperosmotic stress it causes [67,68]. The highly significant and largely inhibitory effects of maternal diabetes on the structure and function of the cytoskeleton indicates that the teratological effects of embryonic hyperglycemia are at least in part mediated by this fairly general insult on cellular integrity.

### Hyperglycemia and metabolism

The preimplantation embryo becomes exposed to hypoxic conditions when entering the uterus [69]. Coincident with the accompanying transition to glycolytic energy metabolism, the mitochondrial cristae become tubular and vesicular, while the reappearance of lamellation of the cristae during early organogenesis is a sign of mitochondrial maturation [70–72]. In agreement, oxidative phosphorylation begins to contribute increasingly to energy metabolism after ED9.5 [73,74]. Embryonic hyperglycemia delays this maturation and induces mitochondrial swelling [75]. In agreement, we observed that maternal hyperglycemia suppressed the expression of many genes involved in mitochondrial function. Perhaps even more importantly, we showed that on ED9.5, glycolytic energy production at ED9.5 still accounts for ~99.5% of total energy production. To place this number in perspective, one has to realize that the oxygen consumption rate at ED9.5 was only ~1% of that of primary hepatocytes, while glycolysis measured as the rate of extracellular acidification exceeded that of primary hepatocytes by ~10-fold in experiments carried out in the same month on the same Seahorse analyzer (EH Gilglioni, unpublished data). These numbers demonstrate that glycolysis is still the predominant energy source at ED9.5 and that the ~30% decline in glycolytic activity and capacity deprived hyperglycemic ED9.5 embryos of a substantial share of their capacity to produce ATP. This finding contrasts with the prevalent hypothesis that the teratogenic effects of hyperglycemia have to be ascribed to the oxidative stress that arises from flooding the embryonic mitochondria with respiratory substrates [76,77], and/or to the activation of the hexosamine pathway and associated inhibition of NADPH regeneration[78], which would compromise the still poorly developed antioxidative defense [79].

### Hyperglycemia and congenital malformations

The association of maternal diabetes and congenital malformations of the cardiovascular and neural systems is quite strong [2,5]. To demonstrate that some of the genes that are differentially regulated by hyperglycemia are indeed causally involved in the development of malformations, one has to interfere with their expression. In this respect, it should be kept in mind that the majority of genes were downregulated on both ED8.5 and ED9.5 (~80% on ED8.5 and 65% on ED9.5). We, therefore, queried the Jackson Laboratory MGD database of genes causing cardiovascular or neural if deficient. This query showed that 20–25% of the genes regulated by hyperglycemia have already been demonstrated to cause heart, neural-tube, or embryonic malformations if deficient. Almost one quarter of these genes are shared between heart and neural tube, in agreement with the fact that these malformations often co-occur [2,4,5]. A similar approach was carried out on ED10.5 embryos from hyperglycemic and control mice [16,17]. In this study, which focused on neural-tube defects, the genes that were affected by maternal diabetes and that caused malformations were far more often part of the Wnt, Hh, Notch, or MAPK signaling pathways than of the cytoskeletal network. These data suggest that the hyperglycemia-induced involvement of the cytoskeleton is no longer prominent at ED10.5. Similarly, the effect of hyperglycemia on mitochondrial structure reportedly disappears with age in the embryo [75].

## Limitations of the Study

The choice of the animal model is obviously crucial for the relevance of the outcome of the study. We used STZ to induce diabetes and insulin pellets to modify the type-1 diabetic state into the more type-2-like diabetes of pregnancy. In agreement with this aim, the dependence on insulin increased with the duration of pregnancy. The genetic non-obese diabetic (NOD) model resembles the STZ model in also being an autoimmune type-1-like model [80], but has not been studied extensively as a diabetes of pregnancy model, possibly because it also produces laterality defects [81,82]. Recently, a type-2-like murine model of diabetic pregnancy was reported in which insulin resistance was induced by feeding mice for 15 weeks a very high-fat (~60 en%) diet [83]. The controls were fed a 10 en% fat diet. However, as we (unpublished observations) and others [84,85] have experienced, a high fat content of the diet (>20 en%), as found in the 5020 (9F) and 5015 mouse diets, appears to be a precondition for the development of cardiovascular and neural tube-closure defects in the offspring, because otherwise similar diets that contain <13.5 en% (e.g. mouse diet 5001) do not produce the malformations. Since the effect of dietary fat is established almost instantaneously [84], diet-induced obesity models of diabetic pregnancy offer no obvious advantages over the established STZ model as modified by Loeken [11]. Another caveat of our study is that we carried out gene expression analysis on only 2 stages of development (ED8.5 and 9.5). These stages, which correspond to very early pregnancy in human (~3 weeks gestation), were considered potentially interesting, because they precede the appearance of malformations. Because we could not select for affected embryos we opted to use a validated mouse model of diabetic pregnancy.

## Supporting Information

S1 Appendix The filled out “ARRIVE” guidelines checklist. (PDF)

S1 Fig Body weight after STZ treatment and linbit implantation. Body weight was measured before STZ treatment (“0”), 14 days after the first STZ injection, on the day of linbit implantation (arrow) and on the indicated days after linbit implantation. Round (N = 4) and diamond (N = 12) symbols represent two different series of female mice. The error bar show SEMs.
(TIF)

S2 Fig H&E stained sections of control and hyperglycemic ED9.5 mouse embryos. Panels A and C: control, normoglycemic embryos; panels B and D: experimental, hyperglycemic embryos. Bar: 100 μm.
(TIF)

S3 Fig Correlation between total RNA input and number of sequenced genes after SOLiD SAGE sequencing. Between ~0.3 and 5.2μg total RNA from 29 hyperglycemic and control embryos was used for SOLiD SAGE deep sequencing. The X-axis indicates the amount of RNA used for cDNA synthesis and sequencing, while the Y-axis indicates the saturation in the number of unique expressed genes for each individual embryo.
(TIF)

S4 Fig Genes differentially expressed in hyperglycemic ED8.5 and ED9.5 embryos associated with cardiovascular and/or neural defects. Panel A: 1024 and 2148 were differentially expressed unique functional genes in embryos from hyperglycemic and control dams on ED8.5 and 9.5, respectively, 876 of which were common to ED8.5 and ED9.5. Panels B-D show Venn diagrams of the number of differentially expressed genes associated with cardiovascular (panel B), neural (panel C), and both cardiovascular and neural malformations in Jackson’s MGD database in ED8.5 (blue), ED9.5 (red), or both ED8.5 and ED9.5 embryos (overlap).
(TIF)

S5 Fig Prevalence of genes that were differentially regulated in hyperglycemic ED8.5 and ED9.5 embryos in the MGD data base. Approximately 45% of the detected differentially expressed sequences represented unique genes at both ED8.5 and ED9.5 (removal of Riken, expressed sequence tags (EST), unassigned and hypothetical genes). Of these, ~21% and ~5% were 5- and 10-fold upregulated, respectively, at ED8.5 and ~14% and ~4%, respectively at ED9.5. Approximately 26% of the differentially regulated genes are associated with cardiovascular, neural, or embryogenesis defects if absent. (MGD data base). Among ≥ 5-fold upregulated genes, this percentage is 23–24% and among ≥ 10-fold upregulated genes 18–20%.
(TIF)

S1 Table Criteria to determine the developmental stage of ED8.5 and ED9.5 embryos. The age of embryos was defined according to the criteria of Kaufman [30].
(DOC)

S2 Table PCR primers used. In all cases, the annealing temperature was 60°C.
(DOC)

S3 Table Unique differentially expressed genes in ED8.5 and ED9.5 hyperglycemic and control embryos, and their presence or absence in the MGD database. All listed genes (1024 at ED8.5 (sheet 1) and 2148 at ED9.5 (sheet 2)) are unique and were >1.5-fold in- or decreased in expression (P_adj_ < 0.05) in hyperglycemic embryos. Columns A to H represent “Gene Name”, “Ensembl Gene”, “Unigene”, “Fold Change”, “Log2 Fold Change”, “P adjusted value”, “Chromosome” and “Gene Description”, respectively. The gray fields indicate that the indicated gene was ≥ 5-fold up- or downregulated and present in the subfield cardiovascular, neural, and embryogenesis defects of the Jackson Laboratory Mouse Genome Database (MGD). Columns L to W represent the normalized number of reads for the hyperglycemic and control embryos at ED8.5 and columns L to AA at ED9.5.
(XLS)

S4 Table Expression levels of genes shown in Fig 5. (DOC)

## References

[1] Becerra JE, Khoury MJ, Cordero JF, Erickson JD (1990) Diabetes mellitus during pregnancy and the risks for specific birth defects: a population-based case-control study. Pediatrics 85: 1–9. 2404255

[2] Kucera J, Lenz W, Maier W (1965) Malformations of the lower limbs and the caudal part of the spinal column in children of diabetic mothers. Ger Med Mon 10: 393–396. 5865553

[3] Passarge E, Lenz W (1966) Syndrome of caudal regression in infants of diabetic mothers: observations of further cases. Pediatrics 37: 672–675. 5930030

[4] Sheffield JS, Butler-Koster EL, Casey BM, McIntire DD, Leveno KJ (2002) Maternal diabetes mellitus and infant malformations. Obstet Gynecol 100: 925–930. 12423854 10.1016/s0029-7844(02)02242-1

[5] Evers IM, de Valk HW, Visser GHA (2004) Risk of complications of pregnancy in women with type 1 diabetes: nationwide prospective study in the Netherlands. BMJ (Clinical research ed) 328: 915. 10.1136/bmj.38043.583160.EE PMC390158 15066886

[6] Kucera J (1971) Rate and type of congenital anomalies among offspring of diabetic women. J Reprod Med 7: 73–82. 5095696

[7] Narchi H, Kulaylat N (2000) Heart disease in infants of diabetic mothers. Images Paediatr Cardiol 2: 17–23. 22368579 PMC3232483

[8] Loeken MR (2005) Current perspectives on the causes of neural tube defects resulting from diabetic pregnancy. Am J Med Genet C Semin Med Genet 135C: 77–87. 15800853 10.1002/ajmg.c.30056

[9] Deuchar EM (1977) Embryonic malformations in rats, resulting from maternal diabetes: preliminary observations. J Embryol Exp Morphol 41: 93–99. 145455

[10] Giavini E, Broccia ML, Prati M, Roversi GD, Vismara C (1986) Effects of streptozotocin-induced diabetes on fetal development of the rat. Teratology 34: 81–88. 3764781 10.1002/tera.1420340111

[11] Phelan SA, Ito M, Loeken MR (1997) Neural tube defects in embryos of diabetic mice: role of the Pax-3 gene and apoptosis. Diabetes 46: 1189–1197. 9200655 10.2337/diab.46.7.1189

[12] Lian Q, Dheen ST, Liao D, Tay SS (2004) Enhanced inflammatory response in neural tubes of embryos derived from diabetic mice exposed to a teratogen. J Neurosci Res 75: 554–564. 14743439 10.1002/jnr.20006

[13] Fu J, Tay SS, Ling EA, Dheen ST (2006) High glucose alters the expression of genes involved in proliferation and cell-fate specification of embryonic neural stem cells. Diabetologia 49: 1027–1038. 16508779 10.1007/s00125-006-0153-3

[14] Morgan SC, Relaix F, Sandell LL, Loeken MR (2008) Oxidative stress during diabetic pregnancy disrupts cardiac neural crest migration and causes outflow tract defects. Birth Defects Res A Clin Mol Teratol 82: 453–463. 10.1002/bdra.20457 18435457 PMC5452612

[15] Fine EL, Horal M, Chang TI, Fortin G, Loeken MR (1999) Evidence that elevated glucose causes altered gene expression, apoptosis, and neural tube defects in a mouse model of diabetic pregnancy. Diabetes 48: 2454–2462. 10580436 10.2337/diabetes.48.12.2454

[16] Salbaum JM, Kappen C (2010) Neural tube defect genes and maternal diabetes during pregnancy. Birth Defects Res A Clin Mol Teratol 88: 601–611. 10.1002/bdra.20680 20564432 PMC3509193

[17] Pavlinkova G, Salbaum JM, Kappen C (2009) Maternal diabetes alters transcriptional programs in the developing embryo. BMC Genomics 10: 274 10.1186/1471-2164-10-274 19538749 PMC2715936

[18] Jiang B, Kumar SD, Loh WT, Manikandan J, Ling EA, et al (2008) Global gene expression analysis of cranial neural tubes in embryos of diabetic mice. J Neurosci Res 86: 3481–3493. 10.1002/jnr.21800 18655203

[19] Vijaya M, Manikandan J, Parakalan R, Dheen ST, Kumar SD, et al (2013) Differential gene expression profiles during embryonic heart development in diabetic mice pregnancy. Gene 516: 218–227. 10.1016/j.gene.2012.12.071 23287646

[20] Cai CL, Liang XQ, Shi YQ, Chu PH, Pfaff SL, et al (2003) Isl1 identifies a cardiac progenitor population that proliferates prior to differentiation and contributes a majority of cells to the heart. Dev Cell 5: 877–889. 14667410 10.1016/s1534-5807(03)00363-0 PMC5578462

[21] Abu-Issa R, Kirby ML (2007) Heart field: from mesoderm to heart tube. Annu Rev Cell Dev Biol 23: 45–68. 17456019 10.1146/annurev.cellbio.23.090506.123331

[22] Karunanayake EH, Hearse DJ, Mellows G (1976) Streptozotocin: its excretion and metabolism in the rat. Diabetologia 12: 483–488. 135705 10.1007/BF01219512

[23] Maksimovic-Ivanic D, Trajkovic V, Miljkovic DJ, Mostarica Stojkovic M, Stosic-Grujicic S (2002) Down-regulation of multiple low dose streptozotocin-induced diabetes by mycophenolate mofetil. Clin Exp Immunol 129: 214–223. 12165076 10.1046/j.1365-2249.2002.01901.x PMC1906457

[24] Jungheim ES, Moley KH (2008) The impact of type 1 and type 2 diabetes mellitus on the oocyte and the preimplantation embryo. Semin Reprod Med 26: 186–195. 10.1055/s-2008-1042957 18302110

[25] Matsumura H, Urasaki N, Yoshida K, Kruger DH, Kahl G, et al (2012) SuperSAGE: powerful serial analysis of gene expression. Methods Mol Biol 883: 1–17. 10.1007/978-1-61779-839-9_1 22589121

[26] Christodoulou DC, Gorham JM, Kawana M, DePalma SR, Herman DS, et al (2011) Quantification of gene transcripts with deep sequencing analysis of gene expression (DSAGE) using 1 to 2 microg total RNA. Curr Protoc Mol Biol: 25B–29. 10.1002/0471142727.mb25b09s93 PMC4139004 21225638

[27] Breyer MD, Bottinger E, Brosius FC 3rd, Coffman TM, Harris RC, et al (2005) Mouse models of diabetic nephropathy. J Am Soc Nephrol 16: 27–45. 15563560 10.1681/ASN.2004080648

[28] Fraser RB, Waite SL, Wood KA, Martin KL (2007) Impact of hyperglycemia on early embryo development and embryopathy: in vitro experiments using a mouse model. Hum Reprod 22: 3059–3068. 17933753 10.1093/humrep/dem318

[29] Shen XH, Han YJ, Yang BC, Cui XS, Kim NH (2009) Hyperglycemia reduces mitochondrial content and glucose transporter expression in mouse embryos developing in vitro. J Reprod Dev 55: 534–541. 19550108 10.1262/jrd.20231

[30] Kaufman MH (1992) The Atlas of Mouse Development. San Diego: Academic Press.

[31] Jorstad TS, Langaas M, Bones AM (2007) Understanding sample size: what determines the required number of microarrays for an experiment? Trends Plant Sci 12: 46–50. 17229587 10.1016/j.tplants.2007.01.001

[32] Huis in 't Veld RA, Willemsen AM, van Kampen AH, Bradley EJ, Baas F, et al (2011) Deep sequencing whole transcriptome exploration of the sigmaE regulon in Neisseria meningitidis. PLoS One 6: e29002 10.1371/journal.pone.0029002 22194974 PMC3240639

[33] Li H, Durbin R (2009) Fast and accurate short read alignment with Burrows-Wheeler transform. Bioinformatics 25: 1754–1760. 10.1093/bioinformatics/btp324 19451168 PMC2705234

[34] Love MI, Huber W, Anders S (2014) Moderated estimation of fold change and dispersion for RNA-seq data with DESeq2. Genome Biol 15: 550 25516281 10.1186/s13059-014-0550-8 PMC4302049

[35] Ekins S, Kirillov E, Rakhmatulin EA, Nikolskaya T (2005) A novel method for visualizing nuclear hormone receptor networks relevant to drug metabolism. Drug Metab Dispos 33: 474–481. 15608136 10.1124/dmd.104.002717

[36] Nikolsky Y, Nikolskaya T, Bugrim A (2005) Biological networks and analysis of experimental data in drug discovery. Drug Discov Today 10: 653–662. 15894230 10.1016/S1359-6446(05)03420-3

[37] Berkopec A (2007) HyperQuick algorithm for discrete hypergeometric distribution. J Discrete Algorithms 5.

[38] Vandesompele J, De Preter K, Pattyn F, Poppe B, Van Roy N, et al (2002) Accurate normalization of real-time quantitative RT-PCR data by geometric averaging of multiple internal control genes. Genome Biol 3: RESEARCH0034. 10.1186/gb-2002-3-7-research0034 PMC126239 12184808

[39] Lambert JF, Benoit BO, Colvin GA, Carlson J, Delville Y, et al (2000) Quick sex determination of mouse fetuses. J Neurosci Methods 95: 127–132. 10752483 10.1016/s0165-0270(99)00157-0

[40] Bracero LA, Cassidy S, Byrne DW (1996) Effect of gender on perinatal outcome in pregnancies complicated by diabetes. Gynecol Obstet Invest 41: 10–14. 8821877 10.1159/000292026

[41] Innes KE, Wimsatt JH (1999) Pregnancy-induced hypertension and insulin resistance: evidence for a connection. Acta Obstet Gynecol Scand 78: 263–284. 10203292

[42] Gittes GK, Rutter WJ (1992) Onset of cell-specific gene expression in the developing mouse pancreas. Proc Natl Acad Sci U S A 89: 1128–1132. 1371010 10.1073/pnas.89.3.1128 PMC48399

[43] Goodner CJ, Freinkel N (1961) Carbohydrate metabolism in pregnancy. IV. Studies on the permeability of the rat placenta to I-131 insulin. Diabetes 10: 383–392. 13707055 10.2337/diab.10.5.383

[44] Smith DE, Gridley T (1992) Differential screening of a PCR-generated mouse embryo cDNA library: glucose transporters are differentially expressed in early postimplantation mouse embryos. Development 116: 555–561. 1289053 10.1242/dev.116.3.555

[45] Takao Y, Akazawa S, Matsumoto K, Takino H, Akazawa M, et al (1993) Glucose transporter gene expression in rat conceptus during high glucose culture. Diabetologia 36: 696–706. 8405736 10.1007/BF00401139

[46] Morita Y, Tsutsumi O, Oka Y, Taketani Y (1994) Glucose transporter GLUT1 mRNA expression in the ontogeny of glucose incorporation in mouse preimplantation embryos. Biochem Biophys Res Commun 199: 1525–1531. 8147898 10.1006/bbrc.1994.1404

[47] Morita Y, Tsutsumi O, Hosoya I, Taketani Y, Oka Y, et al (1992) Expression and possible function of glucose transporter protein GLUT1 during preimplantation mouse development from oocytes to blastocysts. Biochem Biophys Res Commun 188: 8–15. 1417871 10.1016/0006-291x(92)92342-u

[48] Korgun ET, Demir R, Hammer A, Dohr G, Desoye G, et al (2001) Glucose transporter expression in rat embryo and uterus during decidualization, implantation, and early postimplantation. Biol Reprod 65: 1364–1370. 11673251 10.1095/biolreprod65.5.1364

[49] Schmidt S, Hommel A, Gawlik V, Augustin R, Junicke N, et al (2009) Essential role of glucose transporter GLUT3 for post-implantation embryonic development. J Endocrinol 200: 23–33. 10.1677/JOE-08-0262 18948350 PMC2632781

[50] Trocino RA, Akazawa S, Takino H, Takao Y, Matsumoto K, et al (1994) Cellular-tissue localization and regulation of the GLUT-1 protein in both the embryo and the visceral yolk sac from normal and experimental diabetic rats during the early postimplantation period. Endocrinology 134: 869–878. 8299581 10.1210/endo.134.2.8299581

[51] Li R, Thorens B, Loeken MR (2007) Expression of the gene encoding the high-Km glucose transporter 2 by the early postimplantation mouse embryo is essential for neural tube defects associated with diabetic embryopathy. Diabetologia 50: 682–689. 17235524 10.1007/s00125-006-0579-7

[52] Vannucci SJ, Rutherford T, Wilkie MB, Simpson IA, Lauder JM (2000) Prenatal expression of the GLUT4 glucose transporter in the mouse. Dev Neurosci 22: 274–282. 10965149 10.1159/000017451

[53] Chiu TT, Patel N, Shaw AE, Bamburg JR, Klip A (2010) Arp2/3- and cofilin-coordinated actin dynamics is required for insulin-mediated GLUT4 translocation to the surface of muscle cells. Mol Biol Cell 21: 3529–3539. 10.1091/mbc.E10-04-0316 20739464 PMC2954118

[54] Stockli J, Fazakerley DJ, James DE (2011) GLUT4 exocytosis. J Cell Sci 124: 4147–4159. 10.1242/jcs.097063 22247191 PMC3258103

[55] Brozinick JT Jr., Berkemeier BA, Elmendorf JS (2007) "Actin"g on GLUT4: membrane & cytoskeletal components of insulin action. Curr Diabetes Rev 3: 111–122. 18220662 10.2174/157339907780598199 PMC2396947

[56] Charron MJ, Katz EB (1998) Metabolic and therapeutic lessons from genetic manipulation of GLUT4. Mol Cell Biochem 182: 143–152. 9609123

[57] Membrez M, Hummler E, Beermann F, Haefliger JA, Savioz R, et al (2006) GLUT8 is dispensable for embryonic development but influences hippocampal neurogenesis and heart function. Mol Cell Biol 26: 4268–4276. 16705176 10.1128/MCB.00081-06 PMC1489108

[58] Cheng CH, Kikuchi T, Chen YH, Sabbagha NG, Lee YC, et al (2009) Mutations in the SLC2A10 gene cause arterial abnormalities in mice. Cardiovasc Res 81: 381–388. 10.1093/cvr/cvn319 19028722

[59] Dawson PA, Mychaleckyj JC, Fossey SC, Mihic SJ, Craddock AL, et al (2001) Sequence and functional analysis of GLUT10: a glucose transporter in the Type 2 diabetes-linked region of chromosome 20q12-13.1. Mol Genet Metab 74: 186–199. 11592815 10.1006/mgme.2001.3212

[60] Savagner P (2015) Epithelial-mesenchymal transitions: from cell plasticity to concept elasticity. Curr Top Dev Biol 112: 273–300. 10.1016/bs.ctdb.2014.11.021 25733143

[61] Solnica-Krezel L, Sepich DS (2012) Gastrulation: making and shaping germ layers. Annu Rev Cell Dev Biol 28: 687–717. 10.1146/annurev-cellbio-092910-154043 22804578

[62] Scott-Drechsel DE, Rugonyi S, Marks DL, Thornburg KL, Hinds MT (2013) Hyperglycemia slows embryonic growth and suppresses cell cycle via cyclin D1 and p21. Diabetes 62: 234–242. 10.2337/db12-0161 23193186 PMC3526024

[63] Uenishi E, Shibasaki T, Takahashi H, Seki C, Hamaguchi H, et al (2013) Actin dynamics regulated by the balance of neuronal Wiskott-Aldrich syndrome protein (N-WASP) and cofilin activities determines the biphasic response of glucose-induced insulin secretion. J Biol Chem 288: 25851–25864. 10.1074/jbc.M113.464420 23867458 PMC3764791

[64] Kowluru A (2010) Small G proteins in islet beta-cell function. Endocr Rev 31: 52–78. 10.1210/er.2009-0022 19890090 PMC2852207

[65] Teng B, Lukasz A, Schiffer M (2012) The ADF/Cofilin-Pathway and Actin Dynamics in Podocyte Injury. Int J Cell Biol 2012: 320531 10.1155/2012/320531 22190940 PMC3235464

[66] Ishibashi F (2008) High glucose increases phosphocofilin via phosphorylation of LIM kinase due to Rho/Rho kinase activation in cultured pig proximal tubular epithelial cells. Diabetes Res Clin Pract 80: 24–33. 18093681 10.1016/j.diabres.2007.11.004

[67] Di Ciano-Oliveira C, Thirone AC, Szaszi K, Kapus A (2006) Osmotic stress and the cytoskeleton: the R(h)ole of Rho GTPases. Acta Physiol (Oxf) 187: 257–272. 16734763 10.1111/j.1748-1716.2006.01535.x

[68] Thirone AC, Speight P, Zulys M, Rotstein OD, Szaszi K, et al (2009) Hyperosmotic stress induces Rho/Rho kinase/LIM kinase-mediated cofilin phosphorylation in tubular cells: key role in the osmotically triggered F-actin response. Am J Physiol Cell Physiol 296: C463–475. 10.1152/ajpcell.00467.2008 19109524 PMC5047760

[69] Fischer B, Bavister BD (1993) Oxygen tension in the oviduct and uterus of rhesus monkeys, hamsters and rabbits. J Reprod Fertil 99: 673–679. 8107053 10.1530/jrf.0.0990673

[70] Shepard TH, Muffley LA, Smith LT (1998) Ultrastructural study of mitochondria and their cristae in embryonic rats and primate (N. nemistrina). Anat Rec 252: 383–392. 9811216 10.1002/(SICI)1097-0185(199811)252:3<383::AID-AR6>3.0.CO;2-Z

[71] Mackler B, Grace R, Haynes B, Bargman GJ, Shepard TH (1973) Studies of mitochondrial energy systems during embryogenesis in the rat. Arch Biochem Biophys 158: 662–666. 4150124 10.1016/0003-9861(73)90558-4

[72] Mackler B, Grace R, Duncan HM (1971) Studies of mitochondrial development during embryogenesis in the rat. Arch Biochem Biophys 144: 603–610. 4328160 10.1016/0003-9861(71)90367-5

[73] Clough JR, Whittingham DG (1983) Metabolism of [14C]glucose by postimplantation mouse embryos in vitro. J Embryol Exp Morphol 74: 133–142. 6411849

[74] Houghton FD, Thompson JG, Kennedy CJ, Leese HJ (1996) Oxygen consumption and energy metabolism of the early mouse embryo. Mol Reprod Dev 44: 476–485. 8844690 10.1002/(SICI)1098-2795(199608)44:4<476::AID-MRD7>3.0.CO;2-I

[75] Yang X, Borg LA, Eriksson UJ (1995) Altered mitochondrial morphology of rat embryos in diabetic pregnancy. Anat Rec 241: 255–267. 7710141 10.1002/ar.1092410212

[76] Styrud J, Thunberg L, Nybacka O, Eriksson UJ (1995) Correlations between maternal metabolism and deranged development in the offspring of normal and diabetic rats. Pediatr Res 37: 343–353. 7784144 10.1203/00006450-199503000-00015

[77] Eriksson UJ, Borg LA (1993) Diabetes and embryonic malformations. Role of substrate-induced free-oxygen radical production for dysmorphogenesis in cultured rat embryos. Diabetes 42: 411–419. 8432412 10.2337/diab.42.3.411

[78] Horal M, Zhang Z, Stanton R, Virkamaki A, Loeken MR (2004) Activation of the hexosamine pathway causes oxidative stress and abnormal embryo gene expression: involvement in diabetic teratogenesis. Birth Defects Res A Clin Mol Teratol 70: 519–527. 15329829 10.1002/bdra.20056

[79] el-Hage S, Singh SM (1990) Temporal expression of genes encoding free radical-metabolizing enzymes is associated with higher mRNA levels during in utero development in mice. Dev Genet 11: 149–159. 2379326 10.1002/dvg.1020110205

[80] Reed JC, Herold KC (2015) Thinking bedside at the bench: the NOD mouse model of T1DM. Nat Rev Endocrinol 11: 308–314. 10.1038/nrendo.2014.236 25623120 PMC4523382

[81] Morishima M, Ando M, Takao A (1991) Visceroatrial heterotaxy syndrome in the NOD mouse with special reference to atrial situs. Teratology 44: 91–100. 1957268 10.1002/tera.1420440113

[82] Morishima M, Yasui H, Ando M, Nakazawa M, Takao A (1996) Influence of genetic and maternal diabetes in the pathogenesis of visceroatrial heterotaxy in mice. Teratology 54: 183–190. 9122887 10.1002/(SICI)1096-9926(199610)54:4<183::AID-TERA2>3.0.CO;2-2

[83] Wu Y, Wang F, Fu M, Wang C, Quon MJ, et al (2015) Cellular Stress, Excessive Apoptosis, and the Effect of Metformin in a Mouse Model of Type 2 Diabetic Embryopathy. Diabetes 64: 2526–2536. 10.2337/db14-1683 25720389 PMC4477360

[84] Harris MJ, Juriloff DM (2005) Maternal diet alters exencephaly frequency in SELH/Bc strain mouse embryos. Birth Defects Res A Clin Mol Teratol 73: 532–540. 15968625 10.1002/bdra.20170

[85] Kappen C, Kruger C, MacGowan J, Salbaum JM (2011) Maternal diet modulates the risk for neural tube defects in a mouse model of diabetic pregnancy. Reprod Toxicol 31: 41–49. 10.1016/j.reprotox.2010.09.002 20868740 PMC3035722

