# Supplementary figures and images for: Effect of Hyperglycemia on Gene Expression during Early Organogenesis in Mice
Source: PLoS One. 2016 Jul 19;11(7):e0158035. doi: 10.1371/journal.pone.0158035 (PMC4951019; doi:10.1371/journal.pone.0158035)

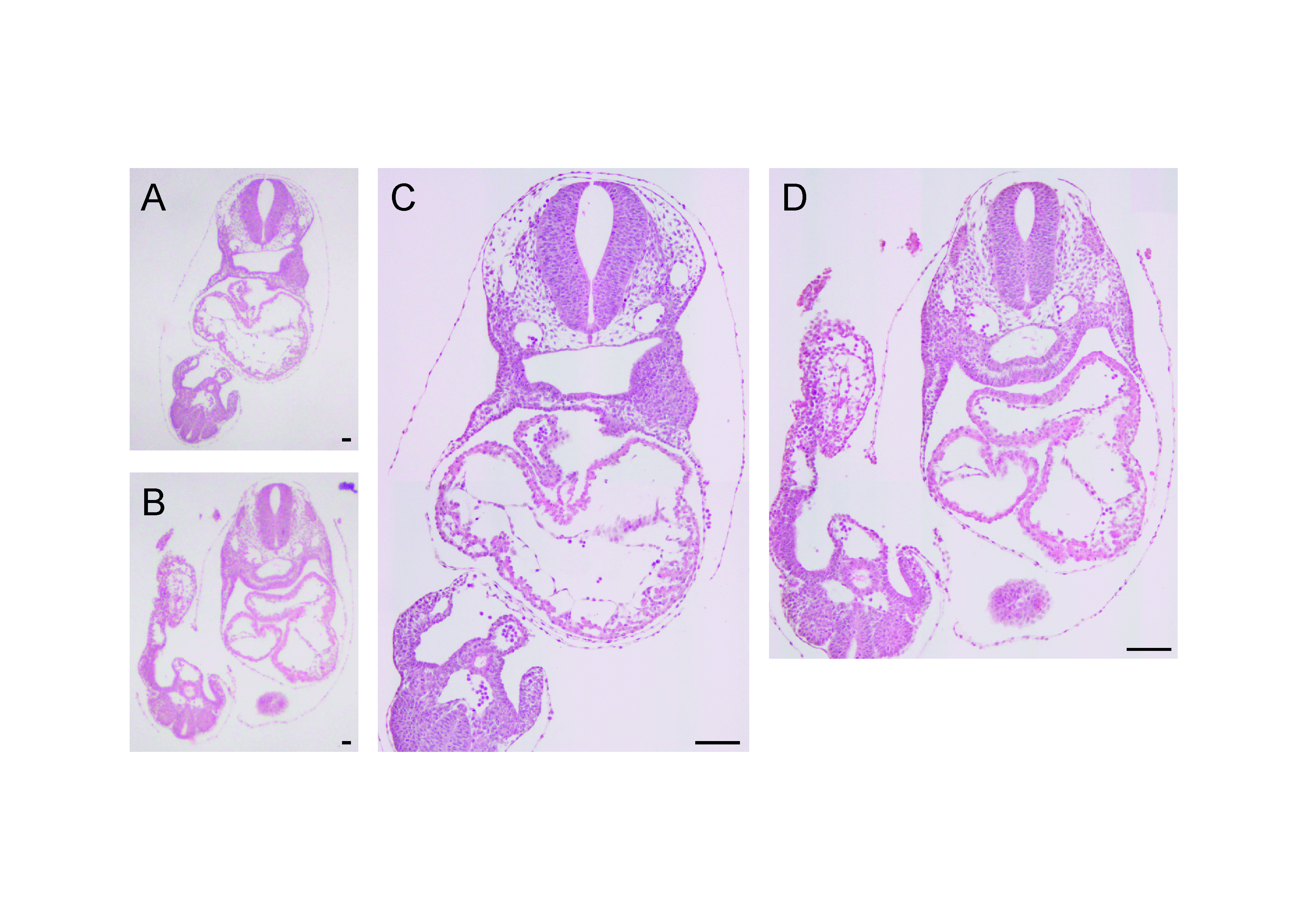

Supplement: S2 Fig — Panels A and C: control, normoglycemic embryos; panels B and D: experimental, hyperglycemic embryos. Bar: 100 μm. (TIF) [file pone.0158035.s003.tif]
