# Supplementary material for: Effect of Hyperglycemia on Gene Expression during Early Organogenesis in Mice
Source: PLoS One. 2016 Jul 19;11(7):e0158035. doi: 10.1371/journal.pone.0158035 (PMC4951019; doi:10.1371/journal.pone.0158035)
Supplement: S1 Table — The age of embryos was defined according to the criteria of Kaufman [30]. (DOC) [file pone.0158035.s007.doc]

**S1 Table. Criteria to determine the developmental stage of ED8.5 and ED9.5 embryos.**

| **Age (ED)** | **# somites** | **Features** |
| --- | --- | --- |
| ED8.5 | ~7 | no “turning” of embryos, neural folds, atrial chamber, allantois |
| ED9.5 | ~20 | Completed turning, closed neural folds, four heart chambers, forelimb, eyes |

The age of embryos was defined according to the criteria of Kaufman.
