# Supplementary material for: Effect of Hyperglycemia on Gene Expression during Early Organogenesis in Mice
Source: PLoS One. 2016 Jul 19;11(7):e0158035. doi: 10.1371/journal.pone.0158035 (PMC4951019; doi:10.1371/journal.pone.0158035)
Supplement: S2 Table — In all cases, the annealing temperature was 60°C. (DOC) [file pone.0158035.s008.doc]

**S2 Table. PCR primers used.**

| **Gene** | **Refseq** | **Forward (5’-3’)** | **Reverse (5’-3’)** |
| --- | --- | --- | --- |
| Actg1 | [NM_009609.2](http://www.ncbi.nlm.nih.gov/nuccore/NM_009609.2) | GATTAAGATCATTGCTCCCCCTGA | GCACCTGCTCAGTCCATCTA |
| Rnaseh2c | [NM_026616.2](http://www.ncbi.nlm.nih.gov/nuccore/NM_026616.2) | GGACTTCGACCGCCTTATCG | TCGCTCCAAGTGGTGAGAAG |
| Cox6c | [NM_053071.2](http://www.ncbi.nlm.nih.gov/nuccore/NM_053071.2) | CGCTGCCTATAAGTTTGGCG | CATAGTTCAGGAGCGCAGGT |
| Trip13 | [NM_027182.2](http://www.ncbi.nlm.nih.gov/nuccore/NM_027182.2) | GACCCTCAGCCCATTGATCT | TGCTGTCCACGTTCTTGTCT |
| Atp5c1 | [NM_001112738.1](http://www.ncbi.nlm.nih.gov/nuccore/NM_001112738.1) | ACCCTTTAAGGTGTGATGGCT | TCGAACTTGGATCCTTGCAGA |
| Tor3a | [NM_023141.2](http://www.ncbi.nlm.nih.gov/nuccore/NM_023141.2) | TGAACCCTACCTGGAACCGA | CCGAGGTTGCTGAGAAAAAGG |
| Mtch1 | [NM_019880.3](http://www.ncbi.nlm.nih.gov/nuccore/NM_019880.3) | AATGCTGGCCCATCCCTTAC | CACACCACTGTACTTGGCCT |
| Mrps23 | [NM_024174.5](http://www.ncbi.nlm.nih.gov/nuccore/NM_024174.5) | TAAGGCCTTTCCACCCCTGA | CACAAACCGCTGACAGGTAG |
| Gtf3c2 | [NM_027901.2](http://www.ncbi.nlm.nih.gov/nuccore/NM_027901.2) | CTGCTGCAGGGAAACAGAAAC | CAACTGCCACTTCCAGGCTA |
| Ndufa6 | [NM_025987.3](http://www.ncbi.nlm.nih.gov/nuccore/NM_025987.3) | AGTACCTCGGTGAAGCCCAT | TGCATTAAGTGCACGGTGTTC |
| Tpm4 | [NM_001001491.1](http://www.ncbi.nlm.nih.gov/nuccore/NM_001001491.1) | CGCGAGAAAGCTGAAGGAGAT | TCGGCTTCGTCAGTGATGTG |
| Marcksl1 | [NM_010807.4](http://www.ncbi.nlm.nih.gov/nuccore/NM_010807.4) | GGCCAACGGACAGGAGAATG | CATCTGTTCCGTTCACGGGT |
| Myosin 1H | [NM_001164573.1](http://www.ncbi.nlm.nih.gov/nuccore/NM_001164573.1) | GCGTTTCCGAGAGAACCTCA | GGCGTAGACATGTGGTGGTA |
| Ubxn8 | [NM_178648.2](http://www.ncbi.nlm.nih.gov/nuccore/NM_178648.2) | CGAGTCTCGGCATTAAAGATCTCA | TTGCCTTTCCCTCACGAGTC |
| Arg1 | [NM_007482.3](http://www.ncbi.nlm.nih.gov/nuccore/NM_007482.3) | CTGGAACCCAGAGAGAGCAT | CTCGAGGCTGTCCTTTTGAGA |
| Grpel1 | [NM_024478.2](http://www.ncbi.nlm.nih.gov/nuccore/NM_024478.2) | CACTGTCGTTCAGGCCTTCTC | GTCTTGTCTGCAGAGGGTGG |
| Pax3 | [NM_001159520.1](http://www.ncbi.nlm.nih.gov/entrez/viewer.fcgi?db=nucleotide&id=226958469) | AACACTGTGCCCTCAGTGAGTTCTAT | ACTCAGGATGCCATCGATGCTGTG |
| Glut4 | [NM_009204.2](http://www.ncbi.nlm.nih.gov/nuccore/NM_009204.2) | CAACTGGACCTGTAACTTCATTGT | ACGGCAAATAGAAGGAAGACGTA |
| Bax | [NM_007527.3](http://www.ncbi.nlm.nih.gov/nuccore/NM_007527.3) | ATGCGTCCACCAAGAAGCTGAG | CCCCAGTTGAAGTTGCCATCAG |
| Cdc20 | [NM_023223.2](http://www.ncbi.nlm.nih.gov/nuccore/NM_023223.2) | TTCGTGTTCGAGAGCGATTTG | ACCTTGGAACTAGATTTGCCAG |
| Cdk1 | [NM_007659.3](http://www.ncbi.nlm.nih.gov/nuccore/NM_007659.3) | ACGGCTTGGATTTGCTCTCA | ACGATCTTCCCCTACGACCA |
| Fgf2 | [NM_008006.2](http://www.ncbi.nlm.nih.gov/nuccore/NM_008006.2) | GCCAACCGGTACCTTGCTAT | GTCCCGTTTTGGATCCGAGT |
| Tab2 | [NM_138667.3](http://www.ncbi.nlm.nih.gov/nuccore/NM_138667.3) | TGGATTTGTAGGCCCTGTGC | CGGATTAAGGCCGGGTGATT |
| Ptpn11 | [NM_001109992.1](http://www.ncbi.nlm.nih.gov/entrez/viewer.fcgi?db=nucleotide&id=158508567) | TTGACTCTCTGACAGACCTGGTG | AGCTTGCTTAACTCTCGAACCCGG |
| Raf1 | [NM_029780.3](http://www.ncbi.nlm.nih.gov/nuccore/NM_029780.3) | AGAGTGCTGTGCAGTGTTCA | CAGGAACGTTTTCCGAGCAA |
| Axin1 | [NM_001159598.1](http://www.ncbi.nlm.nih.gov/entrez/viewer.fcgi?db=nucleotide&id=227430348) | TGACAGCATTGTTGTGGCCTA | CACCTTTTCCACCTTGCCGA |
| Mrto4 | [NM_023536.2](http://www.ncbi.nlm.nih.gov/nuccore/NM_023536.2) | CACTGCCCTCAAGAAAGGTGT | TCTCAGAGTCAGCTGTCATCCTC |
| Psmc4 | [NM_011874.2](http://www.ncbi.nlm.nih.gov/nuccore/NM_011874.2) | CTGGAAGACTATGTGGCCCG | TCTCACGGACAGCCAACATT |
| 18S | NR_003278.3 | TTCGGA ACTGAGGCCATGAT | CGA ACCTCCGACTTTCGTTCT |

In all cases, the annealing temperature was 60°C.
