# Supplementary material for: Effect of Hyperglycemia on Gene Expression during Early Organogenesis in Mice
Source: PLoS One. 2016 Jul 19;11(7):e0158035. doi: 10.1371/journal.pone.0158035 (PMC4951019; doi:10.1371/journal.pone.0158035)
Supplement: S4 Table — (DOC) [file pone.0158035.s010.doc]

**S3 Table. Expression levels of genes shown in Fig. 5**

| **Gene Name** | **SAGE Value±SEM** | **Q-PCR Value±SEM** | **Color** | |
| --- | --- | --- | --- | --- |
| Mrps23 | 400±30 | 1E-05±2E-06 |  |  |
| Gtf3c2 | 1115±54 | 6E-06±1E-06 |  |  |
| Ndufa6 | 792±61 | 9E-06±2E-06 |  |  |
| Tpm4 | 2850±247 | 2E-05±5E-06 |  |  |
| Marcksl1 | 4753±1057 | 6E-05±1E-05 |  |  |
| Myosin 1H | 524±32 | 4E-05±1E-05 |  |  |
| Raf1 | 623±50 | 6E-06±1E-06 |  |  |
| Tab2 | 301±48 | 3E-06±2E-07 |  |  |
| Axin1 | 212±29 | 3E-06±3E-07 |  |  |
| Mrto4 | 474±43 | 2E-05±2E-06 |  |  |
| Psmc4 | 1364±50 | 8E-06±1E-06 |  |  |
| Rnaseh2c | 541±77 | 4E-07±5E-8 |  |  |
| Mtch1 | 4472±170 | 9E-05±2E-05 |  |  |
| Pax3 | 265±32 | 2E-06±2E-07 |  |  |
| Pnpt11 | 505±50 | 3E-05±3E-06 |  |  |
| Bax | 1210±94 | 2E-05±2E-06 |  |  |
| Arg1 | 1284±114 | 3E-06±7E-07 |  |  |
| Cdk1 | 2985±207 | 1E-05±1E-06 |  |  |
| Ubxn8 | 638±77 | 9E-07±2E-07 |  |  |
| Trip13 | 603±52 | 8E-06±6E-07 |  |  |
| Tor3a | 63±9 | 3E-07±3E-8 |  |  |
| Glut4 | 30±4 | 3E-06±3E-7 |  |  |
| Fgf2 | 12±2 | 2E-05±2E-6 |  |  |
| Cdc20 | 325±54 | 2E-03±6E-4 |  |  |
| Actg1 | 928±118 | 2E-04±1E-05 |  |  |
| Atp5c1 | 9624±2882 | 5E-07±5E-8 |  |  |
